# Short-term postsynaptic plasticity facilitates predictive tracking in continuous attractors

**DOI:** 10.3389/fncom.2023.1231924

**Published:** 2023-11-02

**Authors:** Huilin Zhao, Sungchil Yang, Chi Chung Alan Fung

**Affiliations:** Department of Neuroscience, City University of Hong Kong, Kowloon, Hong Kong SAR, China

**Keywords:** computational models, synaptic plasticity, attractor models, predictive coding, NMDA receptors

## Abstract

**Introduction:**

The N-methyl-D-aspartate receptor (NMDAR) plays a critical role in synaptic transmission and is associated with various neurological and psychiatric disorders. Recently, a novel form of postsynaptic plasticity known as NMDAR-based short-term postsynaptic plasticity (STPP) has been identified. It has been suggested that long-lasting glutamate binding to NMDAR allows for the retention of input information in brain slices up to 500 ms, leading to response facilitation. However, the impact of STPP on the dynamics of neuronal populations remains unexplored.

**Methods:**

In this study, we incorporated STPP into a continuous attractor neural network (CANN) model to investigate its effects on neural information encoding in populations of neurons. Unlike short-term facilitation, a form of presynaptic plasticity, the temporally enhanced synaptic efficacy resulting from STPP destabilizes the network state of the CANN by increasing its mobility.

**Results:**

Our findings demonstrate that the inclusion of STPP in the CANN model enables the network state to predictively respond to a moving stimulus. This nontrivial dynamical effect facilitates the tracking of the anticipated stimulus, as the enhanced synaptic efficacy induced by STPP enhances the system's mobility.

**Discussion:**

The discovered STPP-based mechanism for sensory prediction provides valuable insights into the potential development of brain-inspired computational algorithms for prediction. By elucidating the role of STPP in neural population dynamics, this study expands our understanding of the functional implications of NMDAR-related plasticity in information processing within the brain.

**Conclusion:**

The incorporation of STPP into a CANN model highlights its influence on the mobility and predictive capabilities of neural networks. These findings contribute to our knowledge of STPP-based mechanisms and their potential applications in developing computational algorithms for sensory prediction.

## 1. Introduction

The N-methyl-D-aspartate receptor (NMDAR) is a Ca^2+^-permeable, ligand-gated ion channel found mainly in the postsynaptic membrane of neurons, facilitating synaptic transmission (Hunt and Castillo, 2012; Paoletti et al., 2013; Yao et al., 2022). It consists of two GluN1s and two GluN2s (GluN2A-D) and becomes active when two glutamates simultaneously bind to two GluN2s (Monyer et al., 1992; Paoletti et al., 2013; Vyklicky et al., 2014). When activated, it produces a regenerative Ca^2+^ spike, which contributes to signal integration occurring in postsynaptic dendrites (Schiller et al., 2000; Branco and Häusser, 2011; Yang et al., 2015; Noh et al., 2019). The heterogeneity in NMDAR subunits generates the diversity of its properties and functions (Paoletti et al., 2013). NMDARs are crucial for neurotransmission and neuronal communication in the nervous system (Paoletti and Neyton, 2007; Hansen et al., 2018). Their dysfunction resulting from hyperactivity, hypofunction, abnormal subunit expression, altered receptor trafficking or localization may contribute to a variety of neurological diseases and psychiatric conditions (Zhou and Sheng, 2013), such as Huntington's disease (Burtscher et al., 2021), Alzheimer's disease (Liu et al., 2019), depression (Marsden, 2011; Adell, 2020), schizophrenia (Lisman et al., 2008; Adell, 2020; Nakazawa and Sapkota, 2020), and ischemic stroke (Chen et al., 2008). NMDARs also play an essential role in synaptic plasticity, which refers to the strengthening or weakening of electrical postsynaptic responses over time in response to past synaptic activities (Citri and Malenka, 2007). Furthermore, NDMARs profoundly influence synaptic functions that underlie high-level cognitive functions (Paoletti et al., 2013; Bertocchi et al., 2021). For example, selective modulation of subunits of NMDARs impaired long-term potentiation, a typical type of long-term synaptic plasticity, in striatal synapses and thus caused a visuospatial learning deficit (Durante et al., 2019). Another example is the repetitive-training enhanced NMDAR-mediated synaptic transmission in the medial prefrontal cortex that was involved in social memory retrieval (Zhang et al., 2022).

**Figure 1:**
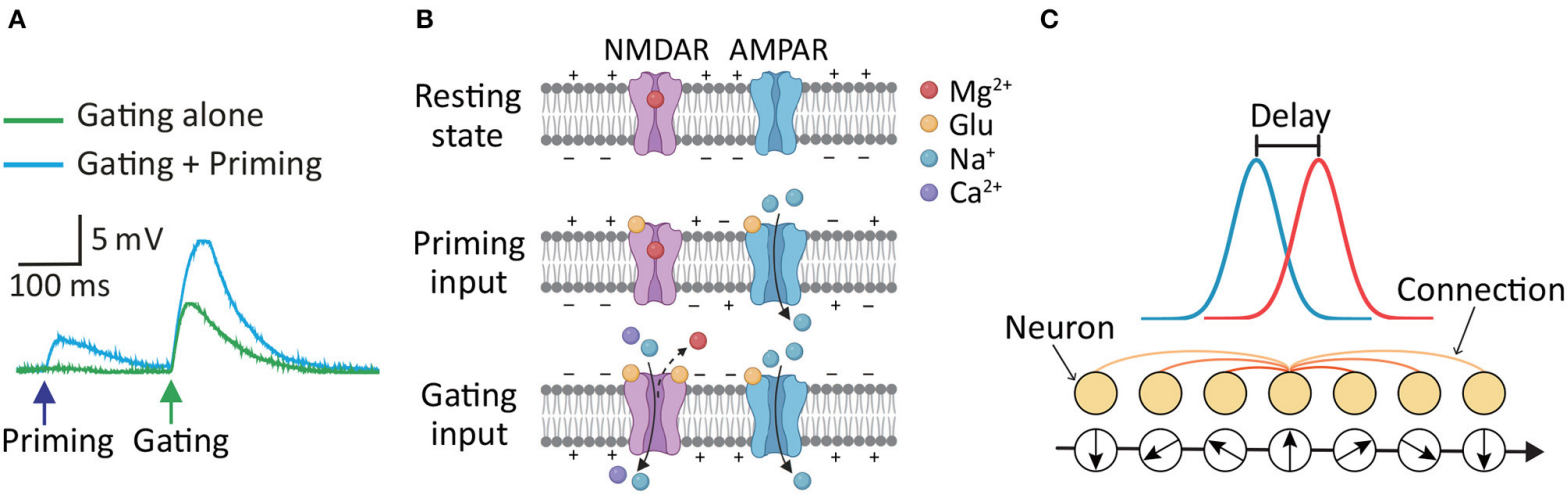
**(A)** Evidence of higher postsynaptic response when priming plus gating (cyan trace) glutamate uncaging is applied than when gating alone (green trace) is applied was observed in a hippocampus dendrite (Yang et al., 2016). **(B)** Schematics of the mechanism of STPP, which is used to interpret the observed increase in postsynaptic response through the actions of receptors on the postsynaptic membrane (Yang et al., 2018). Top: NMDARs and AMPARs are closed in the resting state. Middle: AMPARs are then activated by the priming input, whereas the NMDARs do not open because they are blocked by magnesium ions. However, NMDARs enter the bound-but-blocked state in which the priming input information can be stored for up to 500 ms or longer. Bottom: When a gating input arrives, the postsynaptic membrane is further depolarized and magnesium is removed, shifting the NMDARs from the bound-but-blocked state to the open state. **(C)** Illustration of a CANN that models neural activities involved in continuous stimuli, e.g., head direction. Neurons are arranged in the network in accordance with their preferred stimuli. The excitatory connections among neurons are translationally invariant in the space of stimulus values. The CANN is able to track moving stimuli, i.e., the red bump-shaped curve, but the neuronal activities i.e., the blue bump-shaped curve, always lag behind the stimuli due to the neural response delay (Wu and Amari, 2005).

Although it is widely accepted that NMDAR-mediated Ca^2+^ signaling contributes to long-term synaptic plasticity in postsynaptic neurons (Hunt and Castillo, 2012; Granger and Nicoll, 2014; Volianskis et al., 2015), its effects on short-term synaptic plasticity are gradually being uncovered and studied (Santos et al., 2012; Yang et al., 2014, 2016). Typically, short-term synaptic plasticity is attributed to the difference in the time constants between neuronal signaling and recovery of neurotransmitter availability (Zucker and Regehr, 2002; Mongillo et al., 2008). Neurotransmitter release from presynaptic neurons is primarily responsible for this neurobiological mechanism. Although neuronal plasticity is observed by presynaptic factors for neurotransmitter release, receptors directly modulating the efficacy of postsynaptic currents are situated on the postsynaptic side, which is the postsynaptic NMDARs. According to our previous studies (Yang et al., 2014, 2016, 2018), NMDAR-dependent short-term postsynaptic plasticity (STPP) is proposed to serve as a neurobiological mechanism for signal amplification, particularly in linearly connected circuits such as the hippocampus and cortices. Such signal amplification can be timely achieved through STPP, which enables the faithful transmission of extrinsic information-bearing sensory inputs and the integration of extrinsic sensory inputs (i.e., a priming input) with intrinsic activity (e.g., a brain rhythm or gating input). Unlike the presynaptic mechanism underlying a feedback circuit for signal amplification, this STPP executes a feedforward process to carry out an efficient and timely signal amplification and cascade. One of the observed pieces of evidence showing STPP in a hippocampus dendrite (Yang et al., 2016) is shown in Figure 1A. The postsynaptic response was higher when a second (gating input) glutamate uncaging followed the prior (priming input) uncaging (cyan trace) than when a gating input alone (green trace) was applied. This enhancing effect was eliminated by Ifenprodil, a blocker of GluN2B (Yang et al., 2016). An underlying mechanism (Figure 1B) for short-term signal amplification has been proposed in our earlier publication (Yang et al., 2018). The α-amino-3-hydroxy-5-methyl-4-isoxazolepropionic acid receptor (AMPAR) is another kind of glutamate receptor on the postsynaptic membrane (Diering and Huganir, 2018). NMDARs and AMPARs are closed because the membrane is in the resting state. The AMPARs are then activated by a priming input, i.e., the first glutamate release from the presynaptic neurons, whereas the NMDARs do not open because they are blocked by magnesium ions. However, the input information can be stored via glutamate binding, and the NMDARs enter the bound-but-blocked state for up to 500 ms (or longer) (Yang et al., 2014, 2018). Subsequently, the gating input, i.e., the second glutamate release, is strong enough to further depolarize the postsynaptic membrane, and thus magnesium is removed. Simultaneous membrane depolarization and glutamate binding shift the NMDARs from the bound-but-blocked state to the open state. That is, NMDARs are more likely to open with the previously stored priming input plus gating input than with the gating input alone. The NMDAR-mediated Ca^2+^ current, which is much stronger than the AMPAR current, is thought to contribute to the response enhancement. Additionally, when the priming input was so large to induce NMDAR-mediated Ca^2+^ current, there was no significant enhancement of the second response under the same level of gating input (Figure 1B in Yang et al., 2016). Therefore, STPP depends on the state of postsynaptic NMDARs and the evocation history of postsynaptic currents, but not necessarily the firing history of a *particular* presynaptic neuron. In this regard, STPP has been suggested to enable signal amplification and integration in synaptic transmission, but the role of STPP in the dynamics of neuronal populations remains unknown.

We aim to investigate the possible effects of STPP in neuronal populations on neural information processing. To this end, we introduce the continuous attractor neural network (CANN) as our model for neural information representation (Wu and Amari, 2005; Wu et al., 2016). The CANN is capable of extracting the information encoded by a population of neurons, which allows neural information processing to be analyzed and simulated computationally (Deneve et al., 1999; Wu and Amari, 2005). This model has been successfully used to depict the encoding of continuous stimuli in neural systems, such as movement direction (Georgopoulos et al., 1993), head direction (Ben-Yishai et al., 1995; Zhang, 1996; Stringer S. et al., 2002), the spatial location of objects (Samsonovich and McNaughton, 1997; Stringer S. M. et al., 2002) and spatial integrated information including location, direction and distance (Burak and Fiete, 2009). A CANN is shown in Figure 1C. In the network, a bump-shaped tuning curve (blue curve) represents the neural activities of the population of neurons in response to the external stimuli (red curve) at a given time. Because different neurons can be characterized by their preferred stimuli, e.g., different head directions represent different head-direction (HD) cells, the response curve is a function of the preferred stimuli of the neurons in a population. Among neurons, there are excitatory connections that are translationally invariant in the space of stimulus values. These translation-invariant connections enable a CANN to hold a continuous family of stationary states (attractors). The stationary states of the neural system form a continuous parameter space in which the system is neutrally stable. This property allows the neutral system to track time-varying stimuli smoothly (Wu et al., 2008; Fung et al., 2010). However, the tracking always lags behind the stimulus due to the time needed for neuronal responses and neuronal interactions (Wu and Amari, 2005; Wu et al., 2016). Because CANNs can track moving objects smoothly, they have been used to shed light on the potential neural correlates of effective tracking of moving objects (Zhang, 1996; Fung et al., 2012a; Zhang and Wu, 2012; Mi et al., 2014; Fard et al., 2015). For instance, inhibitory feedback modulations such as short-term depression (STD) of neuronal synapses (Fung et al., 2012a), spike frequency adaptation (SFA) in neural firing (Mi et al., 2014), and negative feedback from a connected network (Zhang and Wu, 2012) have enabled predictive tracking of sensory input with CANNs. A path integration mechanism combined with CANNs has predicted future movement locations (Fard et al., 2015). These neural predictions, i.e., anticipations, of continuously moving objects are powerful strategies for time delay compensation (Nijhawan, 1994; Bassett et al., 2005; Sommer and Wurtz, 2006) and thus maintain effective and efficient perceptual functions and visual/motor control.

In this study, we applied the STPP mechanism to CANNs and examined the tracking dynamics when they tracked moving stimuli. The stimuli could be head directions, object locations or navigation information such as speeds and directions of movements. To simplify our study, we built a one-dimensional CANN model and used head directions as our representative stimuli. Through the simulation experiments and analysis, we found that STPP-induced enhancing effect on neurons around the hillside of synaptic input profile enables CANNs to anticipate the movements of moving stimuli. Unlike the mechanisms for anticipatory tracking based on inhibitory feedback modulations (Fung et al., 2012a; Zhang and Wu, 2012; Mi et al., 2014), STPP is a feedforward modulation driven by the inherent features of NMDARs. Our findings suggest a novel mechanism for anticipatory tracking. The reliable signal transmission enabled by STPP provides a new framework that has the potential to help conceptualize a network mechanism for sensory prediction and develop brain-inspired computational algorithms for prediction.

## 2. Model and simulation

### 2.1. The model

In our study, we used CANNs (Wu and Amari, 2005; Fung et al., 2010) to investigate the influence of STPP (Yang et al., 2016) on the dynamics of neuronal populations. In this model, the dynamics of synaptic input *u*(*x, t*) of neurons with preferred stimuli *x* at time *t* is defined as


(1) $$\begin{array}{l} \tau_{\text{s}}\frac{\partial u\left(x,t\right)}{\partial t}=-u\left(x,t\right)+\left[1+S\left(x,t\right)\right]I^{\text{tot}}\left(x,t\right), \end{array}$$

where τ_s_ ≈ 10ms is the neuronal time constant (Koch et al., 1996). [1 + *S*(*x, t*)] models the efficacy of activity history of postsynaptic neurons in the evocation of postsynaptic input. *I*^tot^(*x, t*), the total input to the neurons from the external input and lateral interactions within the neuronal system, is given by


(2) $$\begin{array}{l} I^{\text{tot}}\left(x,t\right)=\int dx^{\prime }J\left(x,x^{\prime }\right)r\left(x^{\prime },t\right)+I^{\text{ext}}\left(x,t\right). \end{array}$$

*I*^ext^(*x, t*) is the external input, which is defined in the later subsection. *J*(*x, x*′), the excitatory connection between neurons at *x* and *x*′, is given by


(3) $$\begin{array}{l} J\left(x,x^{\prime }\right)=\frac{1}{\sqrt{2\pi }a}\exp\left[-\frac{\text{dist}{\left(x,x^{\prime }\right)}^{2}}{2a^{2}}\right], \end{array}$$

where dist(*x, x*′) describes the distance between *x* and *x*′ depending on the characteristics of the stimulus, which is defined in the following subsection. *a* = 0.5 represents the range of the connection in the preferred stimuli space (Fung et al., 2010) and also controls the tuning width of the attractor states. *r*(*x, t*) is the neuronal response of neurons with preferred stimuli *x* at time *t*. It also encodes the firing rate and is defined as a function of *u*(*x, t*):


(4) $$\begin{array}{l} r\left(x,t\right)=\Theta \left[u\left(x,t\right)\right]\frac{u{\left(x,t\right)}^{2}}{1+\frac{1}{8\sqrt{2\pi }a}k\int dx^{\prime }u{\left(x^{\prime },t\right)}^{2}}, \end{array}$$

where *k* controls the divisive inhibition modeled in the denominator of *r*(*x, t*) (Deneve et al., 1999; Wu and Amari, 2005). Θ is the step function. One should note that *I*^tot^(*x, t*) is a total input integrating the contributions of excitatory and inhibitory signals from a population of neurons regardless of the type of receptors. Also, since the inhibition is divisive, [1 + *S*(*x, t*)] in Equation (1) modulates the excitatory input, which is consistent with the experimental results by Yang et al. (2016).

In Equation (1), *S*(*x, t*) is the enhancing modulation that abstractly models the effect on the synaptic input due to the opening of NMDARs from the bound-but-blocked state. It represents the temporal enhancement due to STPP. Therefore, [1+*S*(*x, t*)]*I*^tot^(*x, t*) models the synaptic input evocation with an enhancement due to STPP (Yang et al., 2016). This term will be further discussed in the Discussion section. The corresponding latent modulation *Q*(*x, t*) of *S*(*x, t*) abstractly models the proportion of NMDARs that enter the bound-but-blocked state. The dynamics of *S*(*x, t*) and *Q*(*x, t*) are defined by


(5) $$\begin{array}{ll} \frac{\partial S\left(x,t\right)}{\partial t} & =-\frac{S\left(x,t\right)}{\tau_{1}}+\alpha Q\left(x,t\right)f_{S}\left[r\left(x,t\right)\right], \end{array}$$

(6) $$\begin{array}{l} \frac{\partial Q\left(x,t\right)}{\partial t}\;=\;-\frac{Q\left(x,t\right)}{\tau_{2}}-\alpha Q\left(x,t\right)f_{S}\left[r\left(x,t\right)\right]\\ \;+\;\beta \left[1-Q\left(x,t\right)\right]f_{Q}\left[I^{\text{tot}}\left(x,t\right)\right], \end{array}$$

where τ_1_ = 50 ms is the time constant of NMDAR (Hestrin et al., 1990), which controls the effective duration of the enhancing effect on postsynaptic input. τ_2_ = 500 ms is the time constant of the latent modulation of STPP (Yang et al., 2018), which controls its effective duration. The parameters α and β, which control the rates of *S*(*x, t*) and *Q*(*x, t*), respectively, are critical for adjusting the effects of STPP. From a physiological point of view, α can be considered the average opening rate of NMDARs and β is the average transition rate of NMDARs from the glutamate-unbound state to the bound-but-blocked state, which is determined by the speed and efficiency of glutamates to bind to NMDARs. *f*_*S*_ and *f*_*Q*_ are activation functions of *S*(*x, t*) and *Q*(*x, t*), respectively. *f*_*S*_ defines the raise of the enhancing modulation *S*(*x, t*). We designed its form based on two considerations: (1) in accordance with the STPP mechanism, the removal of magnesium, which depends on the membrane potential, enhances the excitatory postsynaptic potential by opening NMDARs (Jahr and Stevens, 1990; Vargas-Caballero and Robinson, 2004). Hence, *f*_*S*_ should be sigmoid-shaped based on membrane potential, modeling the magnesium removal efficacy of the postsynaptic membrane. (2) However, in the CANN, the membrane potential is implicit. Based on the study conducted by Latimer's group (Latimer et al., 2019), the average membrane potential can be approximated by the firing rate because they share a rectified-linear relation. In CANNs, *r*(*x, t*) represents the neuronal activity, which corresponds to the firing rate of spiking neurons. In contrast, the synaptic input *u*(*x, t*) integrates neuronal activities from lateral neurons and external input, which has a non-linear relation with the firing rate in the presence of divisive inhibition. Therefore, we chose *r*(*x, t*) as a proxy to represent the average membrane potential for a population of neurons sharing the same preferred stimulus *x*. As a consequence, *f*_*S*_ is defined to be a cumulative distribution function of *r*(*x, t*):


(7) $$f_{S}\left[r\left(x,t\right)\right]=\frac{1}{\sqrt{2\pi }}\int_{-\infty }^{\frac{r\left(x,t\right)-r_{0}}{\sigma_{S}}}dt^{\prime }\exp\left(-\frac{t^{\prime }{\;}^{2}}{2}\right),$$

where *r*_0_ and σ_*S*_ are the mode and scale of its probability density distribution, respectively. *f*_*Q*_ is a function that drives the latent modulation *Q*(*x, t*) depending on the total synaptic input *I*^tot^(*x, t*). Given that insufficient input prevents NMDARs from entering the bound-but-blocked state, *Q*(*x, t*) should raise as *I*^tot^(*x, t*) increases. However, too much input results in strong depolarization and subsequent opening of NMDARs, and moderate input can drive as many NMDARs as possible to enter the bound-but-blocked state (Yang et al., 2016). *Q*(*x, t*) should decrease after *I*^tot^(*x, t*) reaches a moderate value. Nevertheless, strong input would have a chance to leave a portion of NMDARs in the bound-but-blocked state. Therefore, we considered a log-normal distribution with a right skew to strong input to describe the relationship between the probability of entering the bound-but-blocked state and total synaptic input *I*^tot^(*x, t*):


(8) $$\begin{array}{l} f_{Q}\left[I^{\text{tot}}\left(x,t\right)\right]=\frac{1}{I^{\text{tot}}\left(x,t\right)\sigma_{Q}\sqrt{2\pi }}\exp\left[-\frac{|\ln\left[I^{\text{tot}}\left(x,t\right)\right]-\mu_{Q}{|}^{2}}{2\sigma_{Q}^{2}}\right], \end{array}$$

where μ_*Q*_ and σ_*Q*_ are the mode and scale of the natural logarithm of *I*^tot^(*x, t*), respectively.

### 2.2. Simulation experiments

Our aim is to explore the effects of STPP on the tracking dynamics of neuronal populations by using CANNs. To study the dynamics of a CANN with STPP, we built a model using the equations described in the previous subsection and conducted simulation experiments.

#### 2.2.1. Building the model

**Figure 2:**
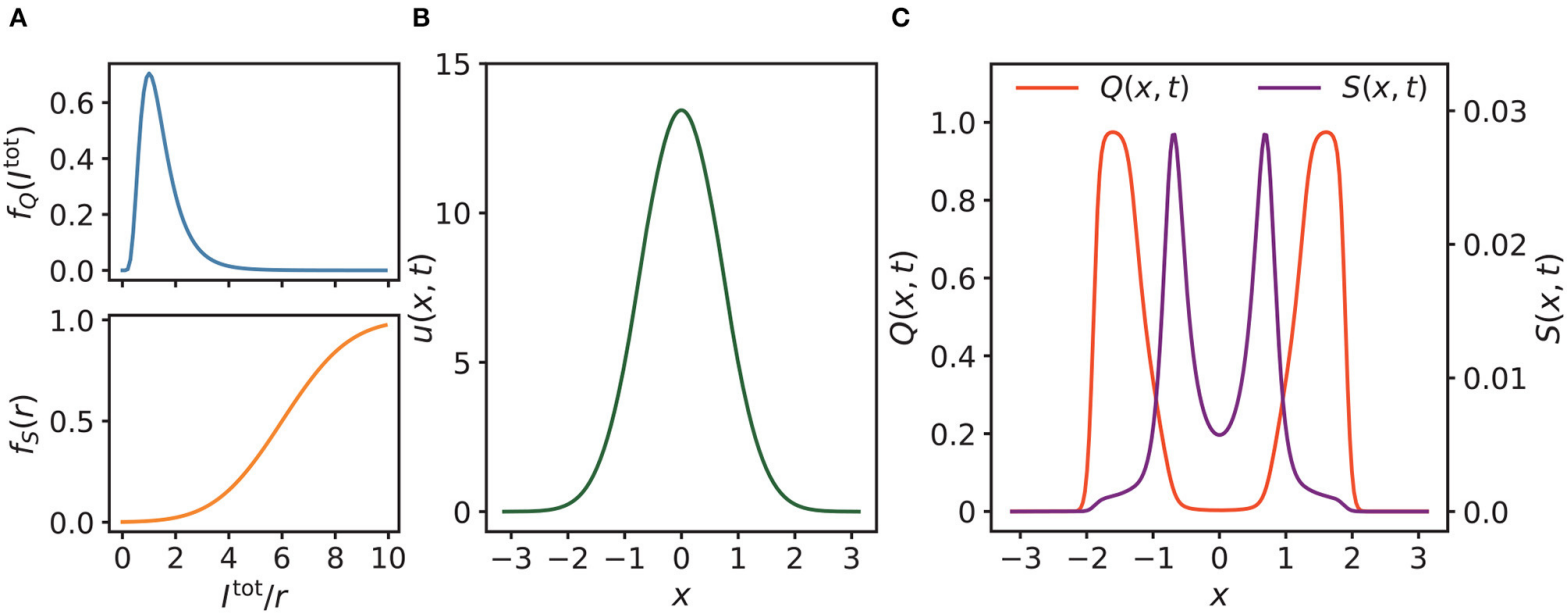
**(A)** Profiles of *f*_*Q*_, *f*_*S*_, where *σ*_*Q*_ = 0.5, *μ*_*Q*_ = 0.25 for *f*_*Q*_, and *r*_0_ = 6, *σ*_*S*_ = 2 for *f*_*S*_. **(B)** As shown, the synaptic input *u*(*x, t*) of neurons is almost Gaussian, where the position of the external input is at 0. **(C)** The corresponding latent and enhancing modulations *Q*(*x, t*) and *S*(*x, t*) of *u*(*x, t*) in **(B)**. As shown, *Q*(*x, t*) and *S*(*x, t*) are initially symmetric with respect to the center of *u*(*x, t*) when there is no external moving drive to *u*(*x, t*). Parameters of **(B, C)**: *a* = 0.5, *k* = 0.5, *A* = 3.0, *α* = 0.02, *β* = 0.1.

For *f*_*Q*_ in Equation (8) and *f*_*S*_ in Equation (7), we assigned the following empirical values: σ_*Q*_ = 0.5, μ_*Q*_ = 0.25, *r*_0_ = 6 and σ_*S*_ = 2. As shown in Figure 2A, *f*_*Q*_ is log-normally distributed, with the latent modulation *Q* increasing most strongly when the total input *I*^tot^(*x, t*) is relatively weak. *f*_*S*_ is sigmoid-shaped, enabling *S*(*x, t*) to be increased at strong membrane potential. Here, the membrane potential is represented by *r*(*x, t*) (Latimer et al., 2019). The profile of the synaptic input *u*(*x, t*) is shown in Figure 2B, which is Gaussian shaped where the center of the external input *I*^ext^(*x, t*) (not plotted) is at 0. The corresponding *Q*(*x, t*) and *S*(*x, t*) are initially symmetric with respect to the center of *u*(*x, t*) when there is no translational separation between *I*^ext^(*x, t*) and *u*(*x, t*) (Figure 2C).

In our simulation experiments, we took the head directions as the example stimuli. Hence, *x* is restricted in the space of −π < *x* ≤ π, and the distance between *x* and *x*′ is in a periodic condition that is calculated by


(9) $$\text{dist}\left(x,x^{\prime }\right)=\{\begin{array}{ll} x-x^{\prime }+2\pi , & \text{if}\left(x-x^{\prime }\right)\leq -\pi ,\\ x-x^{\prime }, & \text{if}-\pi <\left(x-x^{\prime }\right)\leq \pi ,\\ x-x^{\prime }-2\pi , & \text{if}\left(x-x^{\prime }\right)>\pi . \end{array}$$

One should also note that the model we used is a re-scaled model (Fung et al., 2012b). Therefore, the number of neurons is not a factor determining the behavior of the system.

#### 2.2.2. Tracking the external stimulus

To see how the network reacts to a stimulus, we set the external input to be


(10) $$\begin{array}{l} I^{\text{ext}}\left(x\right)=A\exp\left(-\frac{|x-z_{0}{|}^{2}}{4a^{2}}\right), \end{array}$$

where *A* is the magnitude of the input. *z*_0_ is the stimulus position. When considering a continuously moving stimulus, *I*^ext^(*x, t*) is defined as


(11) $$\begin{array}{l} I^{\text{ext}}\left(x,t\right)=A\exp\left[-\frac{|x-z_{0}\left(t\right){|}^{2}}{4a^{2}}\right]. \end{array}$$

Without loss of generality, we considered the stimulus position at time *t* = 0 to be *z*_0_ = 0, and the stimulus to move at a constant angular velocity *v*_ext_ thereafter, i.e., *z*_0_(*t*) = *v*_ext_*t*.

With regard to the tracking behavior simulations, we altered the strength of STPP by adjusting α and β and altered the external factors by adjusting *v*_ext_ and *A* to see how these factors influence the network's tracking performance. To measure the network's tracking performance when it is exposed to a continuously moving stimulus, we used the displacement between the network state and the stimulus position as an index. Note that the network state trails behind the stimulus when *s* and *v*_ext_ have opposite signs, whereas it predicts the position of the stimulus when they have the same sign. The displacement is given by *s* = *z*(*t*)−*z*_0_(*t*), where *z*(*t*) is the center of mass of *u*(*x, t*), which is calculated by


(12) $$\begin{array}{ll} z\left(t\right) & =\tilde{x}+\frac{\int dx\text{dist}\left(x,\tilde{x}\right)u\left(x,t\right)}{\int dxu\left(x,t\right)}, \end{array}$$

(13) $$\tilde{x}=\underset{x}{\text{argmax}}\;u\left(x,t\right)$$

#### 2.2.3. Measuring the intrinsic speed and the anticipatory time

A study (Fung et al., 2015) on dynamical behaviors in neural fields suggested that the models with inhibitory feedback modulations could support spontaneously moving profiles without any persistent external input. Therefore, we asked the following question: in the absence of external input, does a CANN with STPP have intrinsic motion? If the answer is yes, the follow-up question arises: is intrinsic motion caused by STPP? Note that without external input, when the network state becomes translationally unstable, it moves with a constant speed, i.e., intrinsic speed (Fung et al., 2015). To investigate the intrinsic dynamics of a CANN with STPP, we measured the intrinsic speeds, denoted as *v*_int_, of the models when the STPP strength was varied. In the simulations, we first let the system reach its stationary state. After that, we removed the external input and manually shifted *u* in the direction of positive *x* by 2π/200 for every τ_s_. After 100 τ_s_, we terminated all manual intervention and let the system evolve. When the motion of the system state became steady, we recorded the speed of the intrinsic motion as *v*_int_. For the STPP regimes, both α and β were selected from 0 to 0.2 with a step of 0.004.

By analyzing the dynamical properties of the system, the study (Fung et al., 2015) also found that intrinsic motion is the internal drive of anticipation. Hence, after obtaining the intrinsic speeds of the CANNs under various STPP regimes, we considered the following question: if intrinsic motion is present, is it also the internal drive of anticipatory behavior in our model? Therefore, to investigate the causality of anticipation, we measured the maximal anticipatory time *T*_ant_ of the CANNs under the same STPP regimes as those used in the simulations for intrinsic speeds. Here, the anticipatory time τ_ant_ is defined as *s*/*v*_ext_, which is a constant when the moving bump is steady. *T*_ant_ is the maximum of τ_ant_ over a range of *v*_ext_ under a given STPP regime. In the simulations, α and β were also selected from 0 to 0.2 with a step of 0.004. *v*_ext_ was altered from 0 to 0.008 rad/ms with a step of 0.0002. The magnitude *A* of the stimulus is 3.0.

## 3. Results

### 3.1. Tracking behaviors

To observe the tracking behaviors of the CANNs with STPP, we visualized the outlines of the network's motions, first in the presence of an abruptly changing stimulus and then in the presence of a continuously moving stimulus.

#### 3.1.1. In the presence of an abruptly changing stimulus

**Figure 3:**
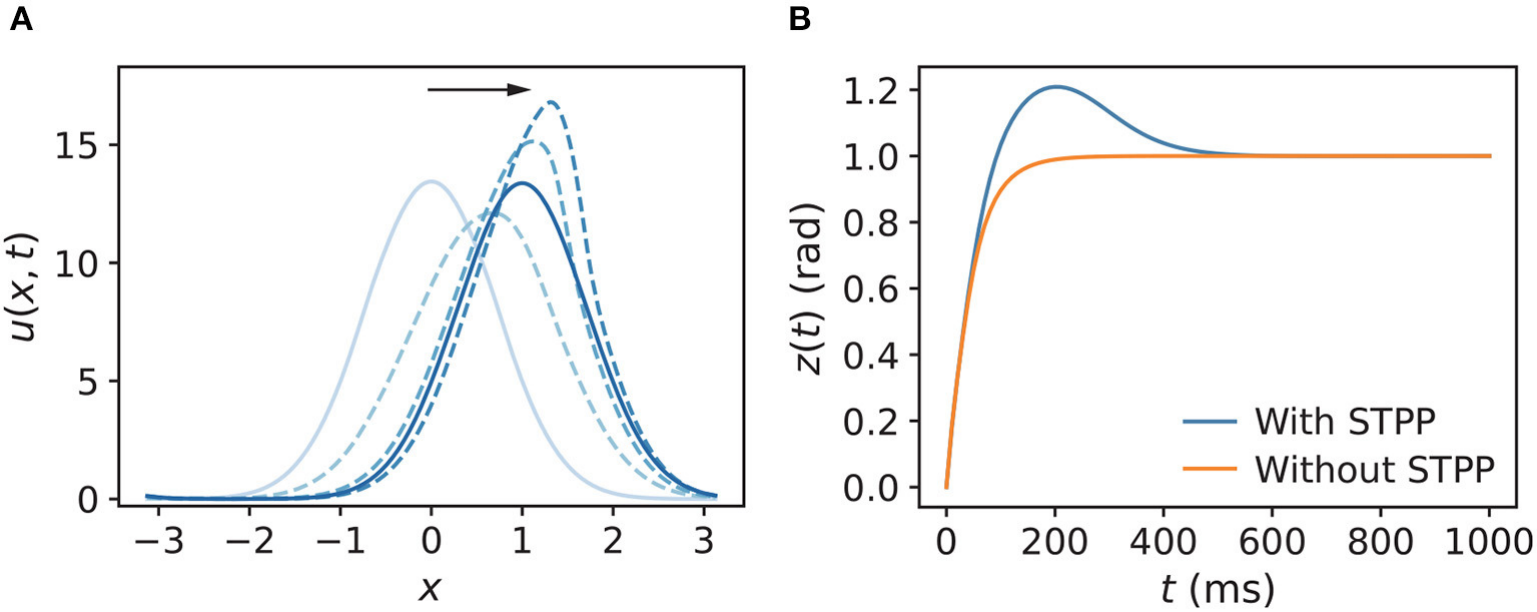
**(A)** Snapshot of *u*(*x, t*) of a CANN with STPP when the stimulus abruptly changes from *z*_0_ = 0 to *z*_0_ = 1. The direction of the motion of *u*(*x, t*) follows the black arrow, and the blue color gradually darkens, representing the new phases over time. The corresponding *z*(*t*) of *u*(*x, t*) is shown in **(B)**. Tracking of an abruptly changing stimulus shows prediction in the CANN with STPP compared to that without STPP. Parameters: *a* = 0.5, *k* = 0.5, *A* = 3.0, model with STPP: *α* = 0.02, *β* = 0.1, model without STPP: *α* = 0, *β* = 0.

We first compared the tracking pattern of a CANN with STPP (α = 0.02, β = 0.1) to that without STPP (α = 0, β = 0) when the stimulus abruptly changed its position *z*_0_. The stimulus position was initially at *z*_0_ = 0 and then shifted to *z*_0_ = 1. As expected, the synaptic input *u*(*x, t*) moved to align the center of mass *z* with the stimulus position *z*_0_ (Figure 3A). Further, the snapshot shows an overshoot of the destination, which is a sign of translational instability of the CANN with STPP. A clearer comparison of *z* was made between a network with STPP and that without STPP (Figure 3B). This instability indicates the potential of STPP for anticipatory tracking.

#### 3.1.2. In the presence of a continuously moving stimulus

**Figure 4:**
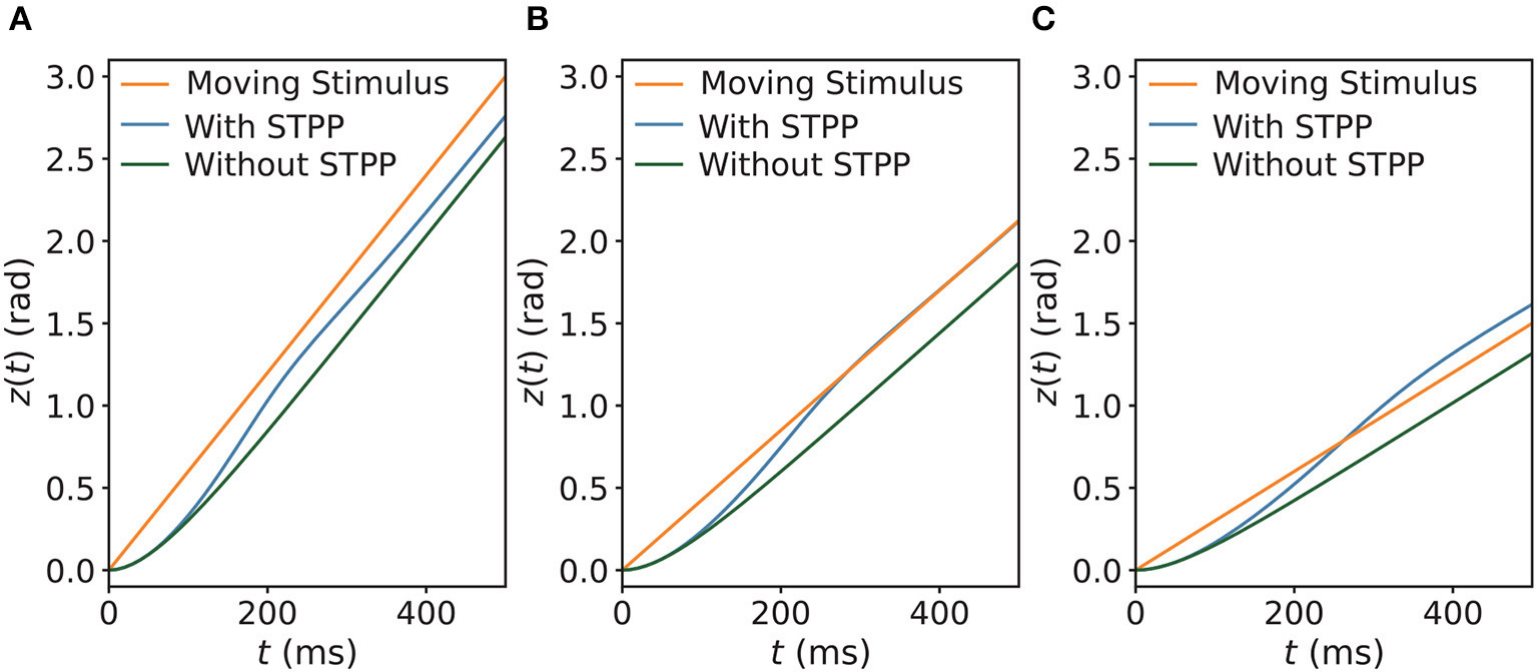
Three comparisons of trackings of a continuously moving stimulus in CANNs with and without STPP. Networks without STPP always exhibit a delay to the moving stimulus, whereas networks with STPP lead with respect to the stimulus. For the same network with STPP, there are mainly three cases of the tracking depending on the angular velocity of stimulus *v*_ext_: **(A)** delayed tracking when *v*_ext_ = 0.006 rad/ms; **(B)** perfect tracking with zero lag when *v*_ext_ = 0.00425 rad/ms; and **(C)** anticipatory tracking with constant time when *v*_ext_ = 0.003 rad/ms. Parameters: *a* = 0.5, *k* = 0.5, *A* = 2.0, models with STPP: *α* = 0.02, *β* = 0.1, models without STPP: *α* = 0, *β* = 0.

Next, we explored the tracking behaviors of the networks in the presence of a continuously moving stimulus by allowing the stimulus to shift with a constant angular velocity *v*_ext_. The CANNs were able to track the moving stimuli by using an approximate speed, which was consistent with how the HD cell system tracks rotating visual cues (Ajabi et al., 2023). The simulations show that the networks without STPP always trailed behind the stimulus regardless of the speed. However, after STPP was applied, three cases of tracking patterns were mainly observed, depending on *v*_ext_: (1) delayed tracking (Figure 4A), (2) perfect tracking with zero lag (Figure 4B), and (3) anticipatory tracking with a constant lead (Figure 4C). Notably, even when there was a delay, the CANN with STPP performed better in tracking the stimulus than pure CANN. In our cases, delayed, perfect and anticipatory trackings occurred in order from fast to slow *v*_ext_. However, delay and alignment may occur in both slow and fast zones, which is discussed in the next subsection. These results suggest that the predictions of the CANNs with STPP depend on the speeds of the stimulus.

### 3.2. Anticipatory tracking

**Figure 5:**
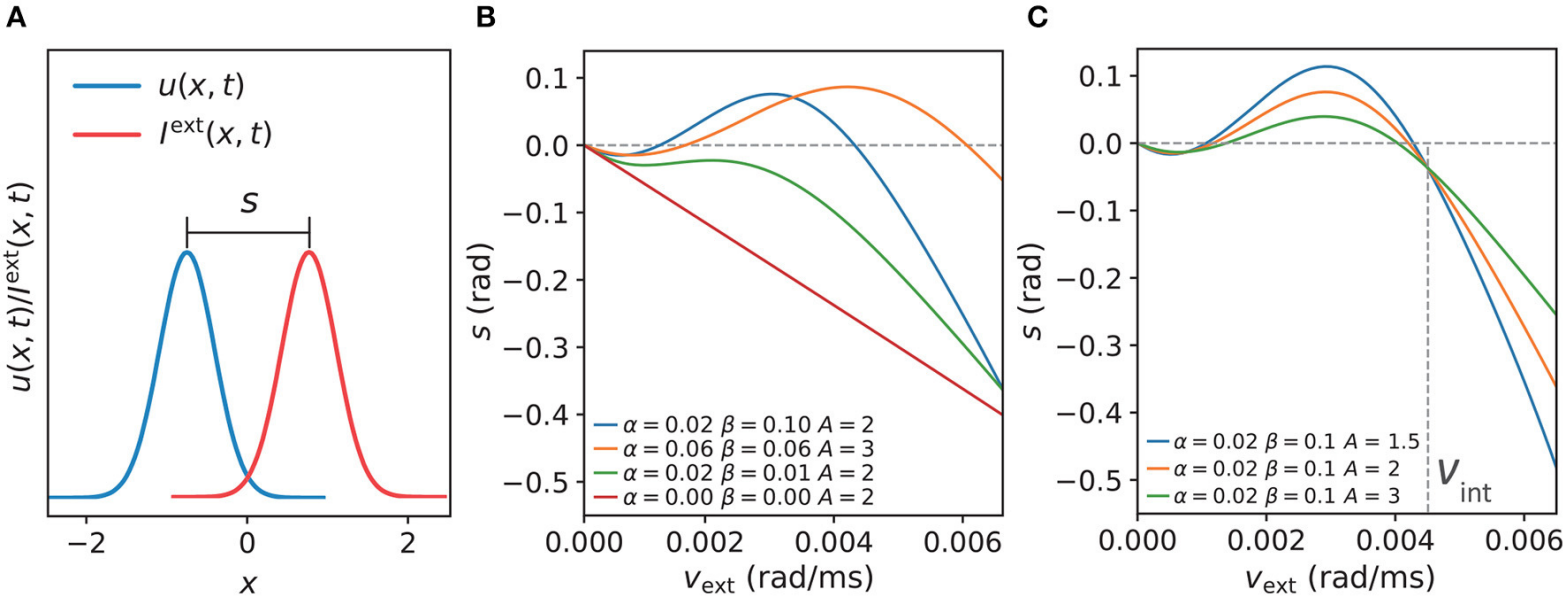
**(A)** The displacement *s* between the network state and stimulus position is defined by *s* = *z*(*t*)−*z*_0_(*t*), which indicates anticipation when *s* and *v*_ext_ have the same signs and delay when *s* and *v*_ext_ have opposite signs. **(B)** Comparisons of *s* of the CANNs at varying speeds *v*_ext_ of moving stimulus with different STPP parameters (α, β). As shown, the network without STPP (α = 0.00, β = 0.00) linearly lags behind the stimulus (gray dashed line at *s* = 0) as *v*_ext_ increases. The network with weak STPP (α = 0.02, β = 0.01) also lags behind the stimulus. However, the networks with certain strengths of STPP, e.g., α = 0.06, β = 0.06 and α = 0.02, β = 0.10, predict the motion of the stimulus within a limited range of *v*_ext_. With respect to the same network (α = 0.02, β = 0.10), the dependency of the anticipatory performance on the intensity of the stimulus (*A*) is shown in **(C)**. These networks have similar patterns, but the weaker the intensity, the greater the anticipation and the larger the velocity range. Moreover, it is notable that these curves converge on a point at a specific velocity where the displacement is independent of the intensity of the stimulus. The velocity of the confluence point is the same as the intrinsic speed *v*_int_ of the network. Parameters: *a* = 0.5, *k* = 0.5.

Next, we examined the dependencies of anticipatory performance on internal (i.e., STPP parameters) and external (i.e., the strength of the stimulus) factors. The displacement *s* is a measure of the tracking performance (see the section titled “Simulation Experiments” for the equation), as shown in Figure 5A. First, we investigated how the STPP parameters affect the model's tracking performance. We compared *s* among different STPP regimes, the results of which indicate that anticipatory tracking is achieved in a certain range of STPP strength. As shown in Figure 5B, the network without STPP (α = 0, β = 0) linearly lagged behind the stimulus (the gray dashed line at *s* = 0). Moreover, when the STPP was relatively weak due to the small scale of parameters, e.g., the dark green line (α = 0.02, β = 0.01), the network failed to make predictions although it showed less lag. In contrast, the networks with strong STPP elicited prediction successfully over a considerable range of angular velocities. For instance, the network with α = 0.02 and β = 0.1 could anticipate stimuli with velocities ranging from 69°/s to 240°/s. A stronger STPP regime (α = 0.06, β = 0.06) facilitated anticipation of a broader range of velocities ranging from 92°/s to 338°/s. Interestingly, we found that at slower velocities, the networks performed with a tiny delay, and the threshold of the velocity for anticipation was related to both the STPP regime and the stimulus (Figures 5B, C). These results reveal the possible implications of STPP for prediction. On the one hand, effective anticipation of stimuli over an extensive range of velocities gives the neural system great flexibility to adapt to a varying stimulus. On the other hand, the correspondence between the range of anticipation-achievable velocities and the diverse intensities of STPP may imply that distinct brain regions or neurons equipped with distinct synaptic plasticity enable diverse anticipatory tracking performance levels.

Second, to understand how the strength of a stimulus affects anticipatory performance, we applied different magnitudes *A* of a stimulus to the same network and compared the results. According to the results shown in Figure 5C, the anticipatory lead was greater and the anticipatory velocity range was larger under the weaker stimulus. Moreover, these traces were confluent at a specific velocity, where the displacement was independent of the intensity of the stimulus. Interestingly, this velocity was the same as the intrinsic speed *v*_int_ of the network, which is described and discussed in the next subsection. In line with the results of natural tracking obtained in an earlier study (Fung et al., 2015), our results also showed confluent behavior, which reveals a certain relationship between intrinsic dynamics and tracking dynamics. That is when the stimulus speed is the same as the intrinsic speed, the displacement is related only to the network itself.

### 3.3. Intrinsic dynamics and anticipatory time

**Figure 6:**
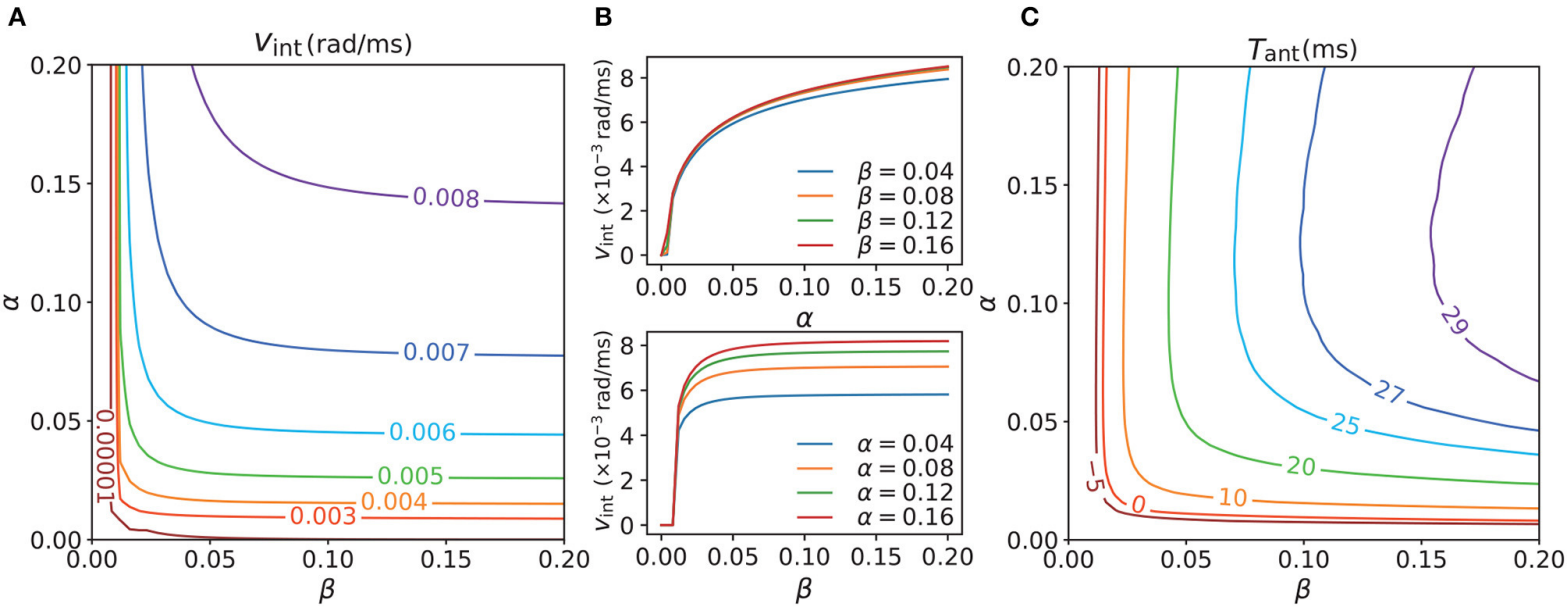
**(A)** Contour map of intrinsic speeds *v*_int_ of CANNs with different STPP regimes. *v*_int_ increases as α or β (or both) increases (increase). **(B)** However, *v*_int_ increases notably along α when β is fixed (top), whereas it becomes almost constant after β reaches a certain level when α is fixed (bottom). Therefore, *v*_int_ is not clearly distinguishable with respect to β whereas it exhibits a clearly hierarchical response to α. It seems that *v*_int_ is more sensitive to α than to β. **(C)** Contour map of the maximal anticipatory time *T*_ant_ of CANNs with the same STPP regimes as those of **(A)**. All of the stimuli possess the same magnitude *A* = 3.0, and *v*_ext_ ∈ [0, 0.008] rad/ms. *T*_ant_ < 0 means delays for all *v*_ext_. The anticipation appears after *v*_int_>0 and *T*_ant_ exhibits a similar trend as *v*_int_, which suggests that intrinsic motion is an internal drive of anticipation and is a necessary condition for it probably. Parameters: *a* = 0.5, *k* = 0.5.

To work out the relationship between the intrinsic dynamics and the tracking dynamics of the network, we next explored the intrinsic dynamics in the absence of a persistent stimulus (we had already tested the tracking dynamics in its presence). The contour map of the intrinsic speeds *v*_int_ under various combinations of α and β is showed in Figure 6A. *v*_int_ increased as α or β (or both) increased. Here, *v*_int_ ≤0.00001 can be considered static because 0.00001 rad/ms is approximately equal to 0.57°/s, which is much lower than the majority of the velocities we recorded. In the region where *v*_int_ ≤0.00001, the network became practically static after manual intervention was terminated. In the moving region, the network became translationally unstable and moved with an intrinsic speed. The intrinsic motion was induced by the strong STPP, whereas the static region was located in parameter regions that lacked STPP or were under weak STPP regimes. Figure 6B shows the separate effects of α and β. *v*_int_ increased notably along α when β was fixed, whereas when α was fixed, it became almost invariant after β reached a relatively high value. Combining the top and bottom sub-figures of Figure 6B reveals that *v*_int_ was not clearly distinguishable with respect to β but exhibited a clearly hierarchical response to α. This behavior implies that *v*_int_ is more sensitive to α than to β.

A certain relationship between the intrinsic dynamics and the tracking dynamics was shown in the previous subsection. In view of this, the question arises: is intrinsic motion the internal drive of anticipatory behavior? To answer this question, we measured the maximal anticipatory time *T*_ant_ of the CANNs under the same STPP regimes as those in Figure 6A to understand the causality of the anticipation. As shown in Figure 6C, the anticipation (*T*_ant_ > 0) appeared after *v*_int_ > 0.00001. Also, the contour map of *T*_ant_ and that of the intrinsic speeds shared a similar trend. These results indicate that stimulus prediction occurs only when there is intrinsic motion and the network is not in an internally static state. Hence, we assume that STPP-induced intrinsic motion is an internal drive of anticipation and a necessary condition for it. The pattern of the maximal anticipatory time map does not perfectly match the intrinsic speed map owing to the nontrivial influence of external input.

### 3.4. Analysis of translational stability of the system

In a dynamical system, spontaneous movement without an external input occurs when the static solution becomes unstable to positional displacement in some parameter regions. To understand the intrinsic dynamics of a CANN with STPP, we studied the translational stability of static solutions of our model. We considered network states with a positional displacement to be


(14) $$u\left(x,t\right)=u_{0}\left(x\right)+u_{1}\left(t\right)\frac{du_{0}\left(x\right)}{dx},$$

(15) $$S\left(x,t\right)=S_{0}\left(x\right)+S_{1}\left(t\right)\left(x-z\right)S_{0}\left(x\right),$$

(16) $$Q\left(x,t\right)=Q_{0}\left(x\right)+Q_{1}\left(t\right)\left(x-z\right)Q_{0}\left(x\right),$$

where *u*_0_(*x*), *S*_0_(*x*), and *Q*_0_(*x*) and *u*_1_(*t*), *S*_1_(*t*), and *Q*_1_(*t*) are the stationary states and the displacements of *u*(*x, t*), *S*(*x, t*), and *Q*(*x, t*), respectively. *z* is the center of mass of *u*_0_(*x*). As derived in Supplementary material,


(17) $$\begin{array}{l} \frac{d}{dt}\left(\begin{array}{l} u_{1}\left(t\right)\\ S_{1}\left(t\right)\\ Q_{1}\left(t\right) \end{array}\right)=\left(\begin{array}{ccc} M_{uu} & M_{uS} & 0\\ M_{Su} & M_{SS} & M_{SQ}\\ M_{Qu} & 0 & M_{QQ} \end{array}\right)\left(\begin{array}{l} u_{1}\left(t\right)\\ S_{1}\left(t\right)\\ Q_{1}\left(t\right) \end{array}\right). \end{array}$$

**Figure 7:**
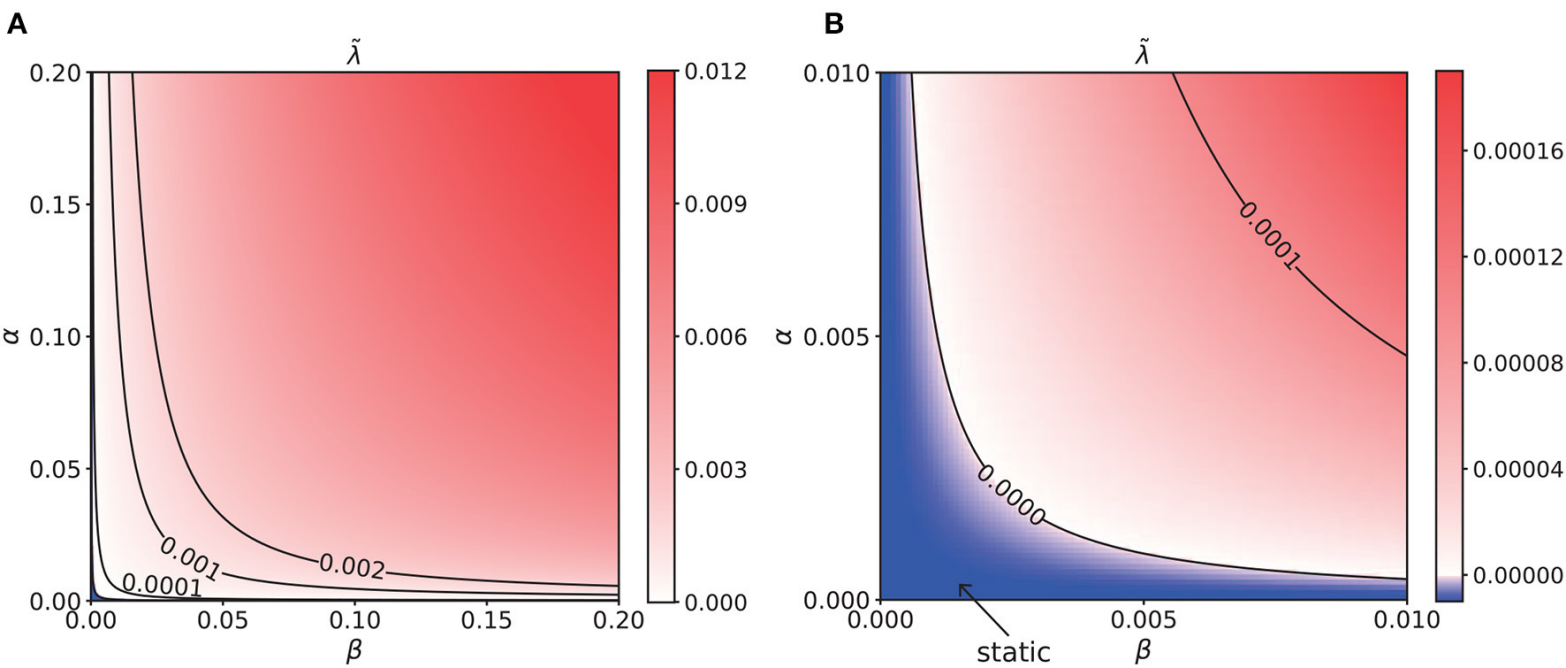
**(A)** Heatmap of the translational stability analysis results, which shares a similar pattern to that of the intrinsic speeds. The unit of α and β is 0.001. **(B)** Enlargement of the bottom left corner of **(A)**. The unit of α and β is 0.0001. The blue region is the static phase of the network where the maximal eigenvalue $\tilde{\lambda }\leq 0$. Elsewhere, it is unstable and spontaneously moves when there is a tiny perturbation. In particular, the parameter region of the intrinsic motion is located in the moving phase of the system. This analysis suggests that the intrinsic dynamics of the network are caused by the translational instability of this system.

For the dynamical system described by Equation (17), the eigenvalue λ of the matrix composed of *M*_ψφ_ (ψ, φ ∈ {*u, S, Q*}) and 0 determines its stability. We calculated the eigenvalues of networks with varying STPP regimes, letting the maximum of the eigenvalues for each regime be denoted as $\tilde{\lambda }$. In the static phase, $\tilde{\lambda }\leq 0$, the network states are stable and static. In the moving phase, $\tilde{\lambda }>0$, the system would diverge due to positional displacement, i.e., perturbation, leading to spontaneously moving bumps. As illustrated in Figure 7, the network states were static when $\tilde{\lambda }\leq 0$ in some STPP regimes, specifically weak STPP regimes. Otherwise, the network state bump moved spontaneously when there was interference. That is, STPP increases the translational instability, i.e., the mobility, of the CANNs. Remarkably, the parameter region of intrinsic motion is in the moving phase of the system. The heatmap of $\tilde{\lambda }$ has a similar pattern to that of the contour map of the intrinsic speeds shown in Figure 6A. Overall, the results indicate that the intrinsic motion of the network is caused by the translational instability of this system, which is induced by STPP.

## 4. Discussion

In this study, we implemented the feedforward signal amplification mechanism of STPP in CANNs. In the model, the STPP effect is modeled by adding two dynamic functions [*Q*(*x, t*) and *S*(*x, t*)] to the original CANN (Wu and Amari, 2005). *Q*(*x, t*) abstractly models the proportion of NDMARs that are in the bound-but-blocked state, while *S*(*x, t*) abstractly models the temporal enhancement dependent on the opening of NMDARs from the bound-but-blocked state. To trigger the bound-but-blocked state, a sufficient but limited synaptic input should be applied (Figure 1B in Yang et al., 2016). To model this effect, we chose the function evoking *Q*(*x, t*), i.e., *f*_*Q*_, to be a bump-shaped log-normal distribution function. On the other hand, when the input to the postsynaptic neuron is uncommonly large, the postsynaptic membrane potential can be sufficient to remove magnesium ions (Jahr and Stevens, 1990; Vargas-Caballero and Robinson, 2004). As a result, the evoked postsynaptic current becomes stronger than that with NMDARs blocked (Yang et al., 2016). To model this magnesium removal efficacy, we chose the cumulative distribution function *f*_*S*_ to trigger the transition from the bound-but-blocked state to the open state. As the average membrane potential can be represented by a firing rate when reasonably assuming that the below-threshold membrane potentials of the postsynaptic neurons are normally distributed around the average value (Latimer et al., 2019), the cumulative distribution function of the firing rate [*r*(*x, t*)] is an appropriate choice to model magnesium removal efficacy on a population of neurons sharing the same preferred stimulus. In summary, functions *f*_*Q*_ and *f*_*S*_ motivated by our previous evidence help visualize the STPP effect.

The additional term [1+*S*(*x, t*)] in Equation (1) models the efficacy of activity history of postsynaptic neurons in the evocation of postsynaptic input. If the neurons were primed, an increase of synaptic input [*S*(*x, t*)*I*^tot^(*x, t*)] was induced in the following glutamate release due to NMDAR-dependent STPP. If there is no STPP, the gain of synaptic input *u*(*x, t*) will become linearly proportional to the total input *I*^tot^(*x, t*) based on CANN (Wu and Amari, 2005). The STPP modulation takes into effect only when NMDARs are primed and sufficient but limited depolarization of membrane is given. Biologically, the total input can be mediated by components on the postsynaptic membrane mainly including AMPARs and inhibitory receptors. When considering the STPP effect, there is an enhancing efficacy on synaptic input with respect to non-plastic total input. Therefore, the modulation is on the evocation of postsynaptic input, instead of the synaptic currents of particular receptors. Despite the inhibition in this model is divisive, nonetheless, the modulation still acts on the cortical and hippocampal networks in the preserved excitatory and inhibitory circuits, as in our previous study (Yang et al., 2016). Although we model the modulating effect on the excitatory synaptic currents, this modeling setting is sufficient to mimic the effect of STPP on CANNs.

Our simulation experiments showed that the implementation of STPP in CANNs successfully enabled anticipatory tracking of a moving stimulus and we also explored the relationship of the model dynamics and anticipation. First, we tested the tracking behaviors of CANNs with STPP when they were exposed to an abruptly changing stimulus and a continuously moving stimulus, and found that this model could predict the future locations of the stimulus. Second, we investigated the intrinsic dynamics of the networks with varying STPP regimes; the results showed that a certain level of STPP supported the intrinsic motion in this model and the intrinsic speed was more sensitive to α. Interestingly, when the angular velocity of the moving stimulus reached the intrinsic speed of the given model, the tracking delay was independent of the strength of the stimulus. Third, by comparing the pattern of the maximal anticipatory time with that of the intrinsic speeds, we found that intrinsic motion is the internal drive of anticipation. Lastly, we analyzed the translational stability of the stationary states of our networks with different STPP parameters. The results implied that strong STPP enhanced the mobility of the system and thus induced intrinsic motion. Taking all the results into account, we noticed that strong α (opening rate of NMDARs) and β (transition rate of NMDARs from the glutamate-unbound state to the bound-but-blocked state) enable anticipation with broader coverage of stimulus velocities and larger maximal anticipatory time. Physiologically, the transition rate of NMDARs to the bound-but-blocked state reflects how fast and efficiently glutamates bind to NMDARs, which is basically dependent on glutamate-NMDAR binding rate and affinity (Paoletti and Neyton, 2007; Singh et al., 2011). Generally, these factors are synaptic specific. Intuitively, the fast opening of NMDARs can help neurons react expeditiously for stimulus chasing, and fast binding can prime NMDARs promptly for further opening. Given that the opening rate (or rise time), the binding rate and affinity are associated with the subunits of NMDAR (Paoletti and Neyton, 2007; Singh et al., 2011; Tu and Kuo, 2014), natural differences in NMDAR subtypes (Buller et al., 1994; Paoletti et al., 2013) would possibly lead to diverse tracking performance for stimuli in various brain regions. In contrast, an abnormal subunit expression of NMDARs may impair perception by the failure of time delay compensation. Collectively, our findings indicate that NMDAR-dependent STPP may underlie sensory prediction, effectively compensating for neural delay and efficiently supporting sensorimotor control.

**Figure 8:**
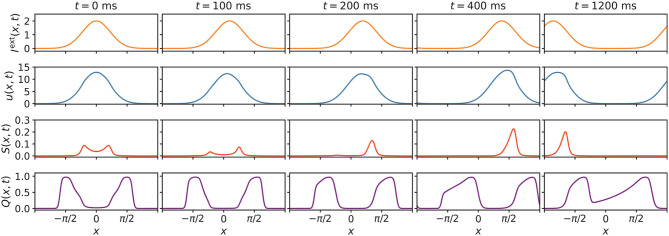
Extracted eight states of *I*^ext^(*x, t*), *u*(*x, t*), *S*(*x, t*), and *Q*(*x, t*) of the network. At the beginning of the external input movement, *u*(*x, t*), *S*(*x, t*) and *Q*(*x, t*) undergo distortions. The biased distortion of *Q*(*x, t*) induces a unimodal distribution of the enhancing modulation [*S*(*x, t*)] with a peak on neighboring neurons that prefer future stimulus positions, thus skewing *u*(*x, t*) toward future positions. This asymmetry results in stronger activation of neurons that favor future positions, which enables this neural system to sense future events. Parameters: *a* = 0.5, *k* = 0.5, *A* = 2.0, α = 0.02, β = 0.1, *v*_ext_ = 0.003 rad/ms.

The underlying mechanism of how STPP can facilitate anticipation can be intuitively understandable. In Figure 8, eight frames of network states were extracted for visualization. From the time-varying states and Equations (1), (4), (5), and (6), we can understand how anticipation occurs. When the stimulus starts moving, the biased distortion of *Q*(*x, t*) induces a unimodal distribution of the enhancing modulation *S*(*x, t*) with the peak on neighboring neurons that prefer future stimulus positions. This consequently skews *u*(*x, t*) toward future positions. This asymmetry leads to stronger activation of the neighboring neurons that exceed the peak of the stimulus, thereby facilitating prediction. In this case, after around 1,200 ms, the states became translationally stable with the same speed as the stimulus; only the locations (not shapes) of the states changed, and thus a constant anticipation time was maintained. Overall, the essence of the anticipatory phenomenon is rooted in the effect of NMDAR-dependent STPP. The information on stimulus positions can be latent in neighboring neurons that are less activated than the stimulus peak-aligned neurons, due to the small input. As the input shifts, the stronger input induces enhanced activation of the neighboring neurons that have stored information. This feedforward effect eventually skews the activation of neurons toward future positions and enables the neural system to sense future events.

Interestingly, despite being a response facilitation mechanism, STPP has the opposite function to that of short-term facilitation (STF). STF refers to a type of short-term synaptic plasticity that boosts the neuronal postsynaptic response as a result of the increased likelihood of neurotransmitter release due to the influx of calcium into the axon terminal after spike generation (Zucker and Regehr, 2002). In a computational study (Fung et al., 2012b) of STF in CANNs, it was discovered that STF improved the behavioral stability of CANNs and served as a noise filter. STF can maintain strong activation of neurons in the active region in accordance with the stimulus position and strengthen interactions among neurons that are tuned to the stimulus. Unlike STPP, this stimulus-specific facilitation stabilizes the network, which makes it incapable of spontaneously moving to anticipate an external input. Inhibitory feedback modulations were considered the basic mechanisms of anticipatory tracking. The modulations include STD, which depresses the activation of postsynaptic neurons by depleting the available resources of neurotransmitters released from presynaptic neurons (Fung et al., 2012a), and SFA, which refers to the reduction of neuronal excitability after prolonged stimulation (Mi et al., 2014). In both STD and SFA, the most active neurons receive the strongest negative feedback to counterbalance their responses, thereby increasing the probability that the locally active network state shifts to the continuously moving stimulus in a sequence. In contrast to inhibitory feedback modulations, which emphasize self-suppression by a feedback system, STPP spotlights the response facilitation on adjacent neurons in a feedforward manner to increase the mobility of the network and achieve anticipatory tracking. Moreover, STPP operates by utilizing the natural properties of NMDARs, which play a significant role in synaptic transmission, rather than relying on recurrent feedback systems, which may involve more auxiliary pathways and systems and may depend on repetitive or prolonged firing (Zucker and Regehr, 2002; Gutkin and Zeldenrust, 2014). It appears that the STPP-dominant feedforward model is simpler and more energy-efficient for anticipatory tracking.

Animal experimental results have suggested that the HD cell system can anchor to local cues, stably tracking rotating cues and maintaining traces when the cues are removed (Taube et al., 1990; Goodridge et al., 1998; Zugaro et al., 2003; Ajabi et al., 2023). However, the mechanism underlying the anchoring and trace maintenance remains unresolved. In 2023, Ajabi's team (Ajabi et al., 2023) found a second dimension in addition to a single angular dimension, namely network gain, to help represent the HD cell system. This discovery indicates that additional information is necessary to fully comprehend how the system adapts to changeable sensory input although the origins of network gain are yet to be identified. They also reported that the system followed the cue's rotation speed to track it and that the system exhibited anticipation. Additionally, the system sustained the drift for some time after the cue was turned off. These findings support the idea that the HD cell system possesses an effective predictive tracking capacity and exhibits spontaneous drift based on experience. To some degree, our model achieved results that were similar to those obtained by these experiments. Our CANN-based model also tracked a moving stimulus with the same velocity (Fung et al., 2010, 2015). Moreover, the incorporation of STPP enabled our model to move spontaneously without persistent external input, i.e., it exhibited intrinsic motion, and showed the anticipatory tracking of a moving stimulus. A very early study (Blair et al., 1997) reported a skew of the peak of the tuning curve toward future positions in the HD cell system rather than just a translational shift to future positions with an invariant bump shape, a finding that was interpreted as suggesting anticipation. This asymmetry can be induced by STPP in our model. As for the anticipatory time, different studies obtained varying average values such as 17 ms (Blair et al., 1997), 23 ms (Taube and Muller, 1998), and 25 ms (Goodridge and Touretzky, 2000) in the anterior thalamus. The anticipatory time of HD cells can vary in different brain regions and even in different cells in the same region (Blair et al., 1997; Taube and Muller, 1998; Goodridge and Touretzky, 2000). In a large STPP parameter region, our model produced anticipations with maximal anticipatory time ranging from 0 to 30 ms, which were of the same order as that observed in animal experiments. Although STD (Fung et al., 2012a) and SFA (Mi et al., 2014) can achieve tracking performance comparable to that obtained by us, the effect of NMDAR-based STPP should not be neglected.

In addition, to prove the robustness of our model to different values of the STPP time constant τ_2_, which represents the lifespan of latent information, we obtained contour maps of *v*_int_ when τ_2_ = 300 ms, τ_2_ = 400 ms, τ_2_ = 600 ms, and τ_2_ = 700 ms through simulations. The results, which are illustrated in Supplementary Figure S1, show that the patterns and achievable ranges of intrinsic speeds in other conditions resemble those obtained with τ_2_ = 500 ms (Figure 6A), demonstrating the robustness of the intrinsic property of our model to anticipation.

Although STPP shows promise as an underlying mechanism for sensory prediction, certain results of our model cannot yet be corroborated by current animal experiments. In our model, the anticipatory time varied with the angular velocities of the stimuli, which usually rose first and then fell. However, the average anticipatory time measured in a population of HD cells in the anterior thalamus tended to be approximately constant over a broad range of head turn velocities (Goodridge and Touretzky, 2000). Differently, some studies reported other thought-provoking issues. The anticipatory time varied among different HD cells in the anterior thalamus (Blair et al., 1997), and the complex results obscured the dependency of the anticipation time on the angular velocity (Bassett et al., 2005). Briefly, due to the considerable variability in real data obtained from the HD cell system, it is challenging to determine the stability of the anticipatory time. More notably, if our proposed mechanism is correct, the differing STPP strength of cells would explain the great complexity of the dependence of the anticipatory time.

In conclusion, our work offers a novel feedforward framework that potentially encodes neural information processing based on STPP in the HD cell system. A consideration of heterogeneous interactions in the neural system shows that NMDAR-dependent STPP may coordinate with other mechanisms, an understanding of which would provide a more comprehensive account of neural information processing. An improved understanding of brain processing and networking may also inspire more computational algorithms for prediction. In the future, assessing the effects of our proposed feedforward framework with other sensory inputs, such as sounds with increasing frequencies and predictable spatial locations, will be a crucial step in validating its applicability.

## Data availability statement

The original contributions presented in the study are included in the article/Supplementary material, further inquiries can be directed to the corresponding authors. The source codes used in this study are available at: https://github.com/fccaa/cann_stpp_2023.

## Author contributions

HZ, SY, and CF conceptualized and designed the study, wrote the paper, and revised the paper. HZ made the computational modeling. HZ and CF performed the data analysis. SY and CF acquired funding. All authors contributed to the article and approved the submitted version.

## References

[B1] Adell A. (2020). Brain NMDA receptors in schizophrenia and depression. Biomolecules 10, 947. 10.3390/biom10060947 32585886 PMC7355879

[B2] Ajabi Z. Keinath A. T. Wei X. Brandon M. P. (2023). Population dynamics of head-direction neurons during drift and reorientation. Nature 615, 892–899. 10.1038/s41586-023-05813-2 36949190 PMC10060160

[B3] Bassett J. P. Zugaro M. B. Muir G. M. Golob E. J. Muller R. U. Taube J. S.. (2005). Passive movements of the head do not abolish anticipatory firing properties of head direction cells. J. Neurophysiol. 93, 1304–1316. 10.1152/jn.00490.2004 15469962

[B4] Ben-Yishai R. Bar-Or R. L. Sompolinsky H. (1995). Theory of orientation tuning in visual cortex. Proc. Nat. Acad. Sci. 92, 3844–3848. 10.1073/pnas.92.9.3844 7731993 PMC42058

[B5] Bertocchi I. Eltokhi A. Rozov A. Chi V. N. Jensen V. Bus T.. (2021). Voltage-independent GluN2a-type NMDA receptor ca^2^+ signaling promotes audiogenic seizures, attentional and cognitive deficits in mice. Commun. Biol. 4, 59. 10.1038/s42003-020-01538-4 33420383 PMC7794508

[B6] Blair H. T. Lipscomb B. W. Sharp P. E. (1997). Anticipatory time intervals of head-direction cells in the anterior thalamus of the rat: implications for path integration in the head-direction circuit. J. Neurophysiol. 78, 145–159. 10.1152/jn.1997.78.1.145 9242269

[B7] Branco T. Häusser M. (2011). Synaptic integration gradients in single cortical pyramidal cell dendrites. Neuron 69, 885–892. 10.1016/j.neuron.2011.02.006 21382549 PMC6420135

[B8] Buller A. Larson H. Schneider B. Beaton J. Morrisett R. Monaghan D.. (1994). The molecular basis of NMDA receptor subtypes: native receptor diversity is predicted by subunit composition. J. Neurosci. 14, 5471–5484. 10.1523/JNEUROSCI.14-09-05471.1994 7916045 PMC6577092

[B9] Burak Y. Fiete I. R. (2009). Accurate path integration in continuous attractor network models of grid cells. PLoS Comput. Biol. 5, e1000291. 10.1371/journal.pcbi.1000291 19229307 PMC2632741

[B10] Burtscher J. Maglione V. Pardo A. D. Millet G. P. Schwarzer C. Zangrandi L.. (2021). A rationale for hypoxic and chemical conditioning in huntington's disease. Int. J. Mol. Sci. 22, 582. 10.3390/ijms22020582 33430140 PMC7826574

[B11] Chen M. Lu T. Chen X. Zhou Y. Chen Q. Feng X.. (2008). Differential roles of NMDA receptor subtypes in ischemic neuronal cell death and ischemic tolerance. Stroke 39, 3042–3048. 10.1161/STROKEAHA.108.521898 18688011

[B12] Citri A. Malenka R. C. (2007). Synaptic plasticity: multiple forms, functions, and mechanisms. Neuropsychopharmacology 33, 18–41. 10.1038/sj.npp.1301559 17728696

[B13] Deneve S. Latham P. E. Pouget A. (1999). Reading population codes: a neural implementation of ideal observers. Nat. Neurosci. 2, 740–745. 10.1038/11205 10412064

[B14] Diering G. H. Huganir R. L. (2018). The AMPA receptor code of synaptic plasticity. Neuron 100, 314–329. 10.1016/j.neuron.2018.10.018 30359599 PMC6214363

[B15] Durante V. de Iure A. Loffredo V. Vaikath N. Risi M. D. Paciotti S.. (2019). Alpha-synuclein targets GluN2a NMDA receptor subunit causing striatal synaptic dysfunction and visuospatial memory alteration. Brain 142, 1365–1385. 10.1093/brain/awz065 30927362

[B16] Fard F. S. Hollensen P. Heinke D. Trappenberg T. P. (2015). Modeling human target reaching with an adaptive observer implemented with dynamic neural fields. Neural Netw. 72, 13–30. 10.1016/j.neunet.2015.10.003 26559472

[B17] Fung C. C. A. Wong K. Wu S. (2012a). “Delay compensation with dynamical synapses,” in Advances in Neural Information Processing Systems, volume 25, eds Pereira F. Burges C. Bottou L. Weinberger K. (Red Hook, NY: Curran Associates, Inc), 640–648.

[B18] Fung C. C. A. Wong K. Y. M. Mao H. Wu S. (2015). Fluctuation-response relation unifies dynamical behaviors in neural fields. Phys. Rev. E 92, 022801. 10.1103/PhysRevE.92.022801 26382448

[B19] Fung C. C. A. Wong K. Y. M. Wang H. Wu S. (2012b). Dynamical synapses enhance neural information processing: gracefulness, accuracy, and mobility. Neural Comput. 24, 1147–1185. 10.1162/NECO_a_00269 22295986

[B20] Fung C. C. A. Wong K. Y. M. Wu S. (2010). A moving bump in a continuous manifold: a comprehensive study of the tracking dynamics of continuous attractor neural networks. Neural Comput. 22, 752–792. 10.1162/neco.2009.07-08-824 19922292

[B21] Georgopoulos A. P. Taira M. Lukashin A. (1993). Cognitive neurophysiology of the motor cortex. Science 260, 47–52. 10.1126/science.8465199 8465199

[B22] Goodridge J. P. Dudchenko P. A. Worboys K. A. Golob E. J. Taube J. S. (1998). Cue control and head direction cells. Behav. Neurosci. 112, 749–761. 10.1037/0735-7044.112.4.749 9733184

[B23] Goodridge J. P. Touretzky D. S. (2000). Modeling attractor deformation in the rodent head-direction system. J. Neurophysiol. 83, 3402–3410. 10.1152/jn.2000.83.6.3402 10848558

[B24] Granger A. J. Nicoll R. A. (2014). Expression mechanisms underlying long-term potentiation: a postsynaptic view, 10 years on. Philos. Trans. R. Soc. B: Biol. Sci. 369, 20130136. 10.1098/rstb.2013.0136 24298139 PMC3843869

[B25] Gutkin B. Zeldenrust F. (2014). Spike frequency adaptation. Scholarpedia 9, 30643. 10.4249/scholarpedia.30643

[B26] Hansen K. B. Yi F. Perszyk R. E. Furukawa H. Wollmuth L. P. Gibb A. J.. (2018). Structure, function, and allosteric modulation of NMDA receptors. J. Gen. Physiol. 150, 1081–1105. 10.1085/jgp.201812032 30037851 PMC6080888

[B27] Hestrin S. Sah P. Nicoll R. A. (1990). Mechanisms generating the time course of dual component excitatory synaptic currents recorded in hippocampal slices. Neuron 5, 247–253. 10.1016/0896-6273(90)90162-9 1976014

[B28] Hunt D. L. Castillo P. E. (2012). Synaptic plasticity of NMDA receptors: mechanisms and functional implications. Curr. Opin. Neurobiol. 22, 496–508. 10.1016/j.conb.2012.01.007 22325859 PMC3482462

[B29] Jahr C. Stevens C. (1990). Voltage dependence of NMDA-activated macroscopic conductances predicted by single-channel kinetics. J. Neurosci. 10, 3178–3182. 10.1523/JNEUROSCI.10-09-03178.1990 1697902 PMC6570236

[B30] Koch C. Rapp M. Segev I. (1996). A brief history of time (constants). Cereb. Cortex 6, 93–101. 10.1093/cercor/6.2.93 8670642

[B31] Latimer K. W. Rieke F. Pillow J. W. (2019). Inferring synaptic inputs from spikes with a conductance-based neural encoding model. Elife 8, e47012. 10.7554/eLife.47012.sa2 31850846 PMC6989090

[B32] Lisman J. E. Coyle J. T. Green R. W. Javitt D. C. Benes F. M. Heckers S.. (2008). Circuit-based framework for understanding neurotransmitter and risk gene interactions in schizophrenia. Trends Neurosci. 31, 234–242. 10.1016/j.tins.2008.02.005 18395805 PMC2680493

[B33] Liu J. Chang L. Song Y. Li H. Wu Y. (2019). The role of NMDA receptors in alzheimer's disease. Front. Neurosci. 13, 43. 10.3389/fnins.2019.00043 30800052 PMC6375899

[B34] Marsden W. (2011). Stressor-induced NMDAR dysfunction as a unifying hypothesis for the aetiology, pathogenesis and comorbidity of clinical depression. Med. Hypotheses 77, 508–528. 10.1016/j.mehy.2011.06.021 21741771

[B35] Mi Y. Fung C. C. A. Wong K. Y. M. Wu S. (2014). “Spike frequency adaptation implements anticipative tracking in continuous attractor neural networks,” in Advances in Neural Information Processing Systems, Volume 27, eds Ghahramani Z. Welling M. Cortes C. Lawrence N. Weinberger K. (Red Hook, NY: Curran Associates, Inc).

[B36] Mongillo G. Barak O. Tsodyks M. (2008). Synaptic theory of working memory. Science 319, 1543–1546. 10.1126/science.1150769 18339943

[B37] Monyer H. Sprengel R. Schoepfer R. Herb A. Higuchi M. Lomeli H.. (1992). Heteromeric NMDA receptors: molecular and functional distinction of subtypes. Science 256, 1217–1221. 10.1126/science.256.5060.1217 1350383

[B38] Nakazawa K. Sapkota K. (2020). The origin of NMDA receptor hypofunction in schizophrenia. Pharmacol. Therap. 205, 107426. 10.1016/j.pharmthera.2019.107426 31629007 PMC6981256

[B39] Nijhawan R. (1994). Motion extrapolation in catching. Nature 370, 256–257. 10.1038/370256b0 8035873

[B40] Noh W. Pak S. Choi G. Yang S. Yang S. (2019). Transient potassium channels: therapeutic targets for brain disorders. Front. Cell. Neurosci. 13, 265. 10.3389/fncel.2019.00265 31263403 PMC6585177

[B41] Paoletti P. Bellone C. Zhou Q. (2013). NMDA receptor subunit diversity: impact on receptor properties, synaptic plasticity and disease. Nat. Rev. Neurosci. 14, 383–400. 10.1038/nrn3504 23686171

[B42] Paoletti P. Neyton J. (2007). NMDA receptor subunits: function and pharmacology. Curr. Opin. Pharmacol. 7, 39–47. 10.1016/j.coph.2006.08.011 17088105

[B43] Samsonovich A. McNaughton B. L. (1997). Path integration and cognitive mapping in a continuous attractor neural network model. J. Neurosci. 17, 5900–5920. 10.1523/JNEUROSCI.17-15-05900.1997 9221787 PMC6573219

[B44] Santos M. D. Mohammadi M. H. Yang S. Liang C. W. Kao J. P. Y. Alger B. E.. (2012). Dendritic hold and read: a gated mechanism for short term information storage and retrieval. PLoS ONE, 7, e37542. 10.1371/journal.pone.0037542 22629416 PMC3358290

[B45] Schiller J. Major G. Koester H. J. Schiller Y. (2000). NMDA spikes in basal dendrites of cortical pyramidal neurons. Nature 404, 285–289. 10.1038/35005094 10749211

[B46] Singh P. Hockenberry A. J. Tiruvadi V. R. Meaney D. F. (2011). Computational investigation of the changing patterns of subtype specific NMDA receptor activation during physiological glutamatergic neurotransmission. PLoS Comput. Biol. 7, e1002106. 10.1371/journal.pcbi.1002106 21738464 PMC3127809

[B47] Sommer M. A. Wurtz R. H. (2006). Influence of the thalamus on spatial visual processing in frontal cortex. Nature 444, 374–377. 10.1038/nature05279 17093408

[B48] Stringer S. Trappenberg T. Rolls E. Araujo I. (2002). Self-organizing continuous attractor networks and path integration: one-dimensional models of head direction cells. Netw. Comput. Neural Syst. 13, 217–242. 10.1080/net.13.2.217.242 12061421

[B49] Stringer S. M. Rolls E. T. Trappenberg T. P. de Araujo I. E. T. (2002). Self-organizing continuous attractor networks and path integration: two-dimensional models of place cells. Netw. Comput. Neural Syst. 13, 429–446. 10.1088/0954-898X_13_4_301 12463338

[B50] Taube J. Muller R. Ranck J. (1990). Head-direction cells recorded from the postsubiculum in freely moving rats. II. effects of environmental manipulations. J. Neurosci. 10, 436–447. 10.1523/JNEUROSCI.10-02-00436.1990 2303852 PMC6570161

[B51] Taube J. S. Muller R. U. (1998). Comparisons of head direction cell activity in the postsubiculum and anterior thalamus of freely moving rats. Hippocampus 8, 87–108. 10.1002/(SICI)1098-1063(1998)8:2&lt;87::AID-HIPO1&gt;3.0.CO;2-4 9572715

[B52] Tu Y.-C. Kuo C.-C. (2014). The differential contribution of GluN1 and GluN2 to the gating operation of the NMDA receptor channel. Pflugers Arch¨. 467, 1899–1917. 10.1007/s00424-014-1630-z 25339225

[B53] Vargas-Caballero M. Robinson H. P. C. (2004). Fast and slow voltage-dependent dynamics of magnesium block in the NMDA receptor: the asymmetric trapping block model. J. Neurosci. 24, 6171–6180. 10.1523/JNEUROSCI.1380-04.2004 15240809 PMC6729657

[B54] Volianskis A. France G. Jensen M. S. Bortolotto Z. A. Jane D. E. Collingridge G. L.. (2015). Long-term potentiation and the role of n-methyl-d-aspartate receptors. Brain Res. 1621, 5–16. 10.1016/j.brainres.2015.01.016 25619552 PMC4563944

[B55] Vyklicky V. Korinek M. Smejkalova T. Balik A. Krausova B. Kaniakova M.. (2014). Structure, function, and pharmacology of NMDA receptor channels. Physiol. Res. 63, S191-9203. 10.33549/physiolres.932678 24564659

[B56] Wu S. Amari S. (2005). Computing with continuous attractors: stability and online aspects. Neural Comput. 17, 2215–2239. 10.1162/0899766054615626 16105223

[B57] Wu S. Hamaguchi K. Amari S.-I. (2008). Dynamics and computation of continuous attractors. Neural Comput. 20, 994–1025. 10.1162/neco.2008.10-06-378 18085986

[B58] Wu S. Wong K. Y. M. Fung C. C. A. Mi Y. Zhang W. (2016). Continuous attractor neural networks: candidate of a canonical model for neural information representation. F1000Res. 5, 156. 10.12688/f1000research.7387.1 26937278 PMC4752021

[B59] Yang S. Chung J. Jin S. H. Bao S. Yang S. (2018). A circuit mechanism of time-to-space conversion for perception. Hear. Res. 366, 32–37. 10.1016/j.heares.2018.05.008 29804722

[B60] Yang S. Santos M. D. Tang C.-M. Kim J. G. Yang S. (2016). A postsynaptic role for short-term neuronal facilitation in dendritic spines. Front. Cell. Neurosci 10, 224. 10.3389/fncel.2016.00224 27746721 PMC5043053

[B61] Yang S. Tang C.-M. Yang S. (2015). The shaping of two distinct dendritic spikes by a-type voltage-gated k^+^ channels. Front. Cell. Neurosci. 9, 469. 10.3389/fncel.2015.00469 26696828 PMC4673864

[B62] Yang S. Yang S. Moreira T. Hoffman G. Carlson G. C. Bender K. J.. (2014). Interlamellar CA1 network in the hippocampus. Proc. Nat. Acad. Sci. 111, 12919–12924. 10.1073/pnas.1405468111 25139992 PMC4156755

[B63] Yao L. Rong Y. Ma X. Li H. Deng D. Chen Y.. (2022). Extrasynaptic NMDA receptors bidirectionally modulate intrinsic excitability of inhibitory neurons. J. Neurosci. 42, 3066–3079. 10.1523/JNEUROSCI.2065-21.2022 35197319 PMC8994549

[B64] Zhang K. (1996). Representation of spatial orientation by the intrinsic dynamics of the head-direction cell ensemble: a theory. J. Neurosci. 16, 2112–2126. 10.1523/JNEUROSCI.16-06-02112.1996 8604055 PMC6578512

[B65] Zhang W. Wu S. (2012). Neural information processing with feedback modulations. Neural Comput. 24, 1695–1721. 10.1162/NECO_a_00296 22428598

[B66] Zhang Y. Xing B. Li Y. Yan C. Gao W. (2022). NMDA receptor-mediated synaptic transmission in prefrontal neurons underlies social memory retrieval in female mice. Neuropharmacology 204, 108895. 10.1016/j.neuropharm.2021.108895 34813859 PMC8688302

[B67] Zhou Q. Sheng M. (2013). NMDA receptors in nervous system diseases. Neuropharmacology 74, 69–75. 10.1016/j.neuropharm.2013.03.030 23583930

[B68] Zucker R. S. Regehr W. G. (2002). Short-term synaptic plasticity. Annu. Rev. Physiol. 64, 355–405. 10.1146/annurev.physiol.64.092501.114547 11826273

[B69] Zugaro M. B. Arleo A. Berthoz A. Wiener S. I. (2003). Rapid spatial reorientation and head direction cells. J. Neurosci 23, 3478–3482. 10.1523/JNEUROSCI.23-08-03478.2003 12716956 PMC6742342

