# Supplementary material for: Short-term postsynaptic plasticity facilitates predictive tracking in continuous attractors
Source: Front Comput Neurosci. 2023 Nov 2;17:1231924. doi: 10.3389/fncom.2023.1231924 (PMC10652417; doi:10.3389/fncom.2023.1231924)
Supplement: Supplementary file 1 [file Data_Sheet_1.PDF]

frontiers

Supplementary Material

1 TRANSLATIONAL STABILITY OF THE DYNAMICAL SYSTEM

In order to study the translational stability issue of static solutions of CANN with STPP. We first looked for the stationary states by considering temporal derivatives in Equations (1), (5) and (6) to be zero and substituted the results into Equations (2) and (4), then we had

$$u_0(x) = [1 + S_0(x)]\, I_0^{\text{tot}}(x), \tag{S1}$$

$$S_0(x) = \tau_1 \alpha Q_0(x)\, f_S[r_0(x)], \tag{S2}$$

$$Q_0(x) = \tau_2 \beta [1 - Q_0(x)]\, f_Q\left[I_0^{\text{tot}}(x)\right] - \tau_2 \alpha Q_0(x)\, f_S[r_0(x)], \tag{S3}$$

$$I_0^{\text{tot}}(x) = \int dx' J(x, x')\, r_0(x'), \tag{S4}$$

$$r_0(x) = \frac{u_0(x)^2}{1 + \frac{1}{8\sqrt{2\pi}a} k \int dx' u_0(x')^2}. \tag{S5}$$

Since the functional forms were complicated, the stationary states were solved by numerical methods through simulations.

Next, we considered the network states with a small positional displacement to be

$$u(x,t) = u_0(x) + u_1(t)\frac{du_0(x)}{dx}, \tag{S6}$$

$$S(x,t) = S_0(x) + S_1(t)(x - z)\, S_0(x), \tag{S7}$$

$$Q(x,t) = Q_0(x) + Q_1(t)(x - z)\, Q_0(x), \tag{S8}$$

where $u_0(x)$, $S_0(x)$, and $Q_0(x)$ and $u_1(t)$, $S_1(t)$, and $Q_1(t)$ are the stationary states and the displacements of $u(x,t)$, $S(x,t)$, and $Q(x,t)$, respectively. z is the center of mass of $u_0(x)$.

Then the function $r(x,t)$ became

$$\begin{aligned} r(x,t) &= \frac{u_0(x)^2 + 2u_0(x)\, u_1(t)\frac{du_0(x)}{dx}}{1 + \frac{1}{8\sqrt{2\pi}a} k \int dx' \left[u_0(x')^2 + 2u_0(x')\, u_1(t)\frac{du_0(x')}{dx}\right]} \\ &= \frac{u_0(x)^2 + 2u_0(x)\, u_1(t)\frac{du_0(x)}{dx}}{1 + \frac{1}{8\sqrt{2\pi}a} k \int dx' u_0(x')^2} \\ &= r_0(x) + \frac{2u_0(x)\, u_1(t)}{B}\frac{du_0(x)}{dx}, \end{aligned} \tag{S9}$$

$$B = 1 + \frac{1}{8\sqrt{2\pi}a} k \int dx' u_0(x')^2. \tag{S10}$$

The function $I^{\text{tot}}(x,t)$ became

$$
\begin{aligned}
I^{\text{tot}}(x,t) &= \int dx' J(x,x')\, r(x',t) \\
&= \int dx' J(x,x') \left[r_0(x) + \frac{2u_0(x)\, u_1(t)}{B} \frac{du_0(x)}{dx} \right] \\
&= \int dx' J(x,x')\, r_0(x') + \frac{2u_1(t)}{B} \int dx' J(x,x')\, u_0(x') \frac{du_0(x')}{dx} \\
&= I_0^{\text{tot}}(x) + \frac{2u_1(t)}{B} \int dx' J(x,x')\, u_0(x') \frac{du_0(x')}{dx}.
\end{aligned} \tag{S11}
$$

For Equation (1), we had

$$
\begin{aligned}
\tau_{\text{s}} \frac{du_1(t)}{dt} \frac{du_0(x)}{dx} &= -u_0(x) - u_1(t) \frac{du_0(x)}{dx} + \left[1 + S_0(x) + S_1(t)(x-z) S_0(x)\right] \\
&\quad \times \int dx' J(x,x') \left[r_0(x') + \frac{2u_0(x')\, u_1(t)}{B} \frac{du_0(x')}{dx} \right] \\
&= \cancel{-u_0(x)} - u_1(t) \frac{du_0(x)}{dx} + \cancel{\left[1 + S_0(x)\right] \int dx' J(x,x')\, r_0(x)} \\
&\quad + 2\frac{u_1(t)}{B} \left[1 + S_0(x)\right] \int dx' J(x,x')\, u_0(x') \frac{du_0(x')}{dx} \\
&\quad + S_1(t)(x-z) S_0(x) \int dx' J(x,x')\, r_0(x') \\
&\quad + 2\frac{u_1(t)}{B} S_1(t)(x-z) S_0(x) \int dx' J(x,x')\, u_0(x') \frac{du_0(x')}{dx}.
\end{aligned} \tag{S12}
$$

By gathering odd terms, we had

$$
\begin{aligned}
\tau_{\text{s}} \frac{du_1(t)}{dt} \frac{du_0(x)}{dx} &= -u_1(t) \frac{du_0(x)}{dx} + 2\frac{u_1(t)}{B} \left[1 + S_0(x)\right] \int dx' J(x,x')\, u_0(x') \frac{du_0(x')}{dx} \\
&\quad + S_1(t)(x-z) S_0(x) \int dx' J(x,x')\, r_0(x'),
\end{aligned} \tag{S13}
$$

$$
\begin{aligned}
\frac{du_1(t)}{dt} &= -u_1(t) \frac{1}{\tau_{\text{s}}} \Bigg\{ 1 - \frac{2}{B} \times \frac{1}{\int dx' \left[\frac{du_0(x')}{dx}\right]^2} \\
&\quad \times \int dx \frac{du_0(x)}{dx} \left[1 + S_0(x)\right] \int dx' J(x,x')\, u_0(x') \frac{du_0(x')}{dx} \Bigg\} \\
&\quad + S_1(t) \frac{1}{\tau_{\text{s}}} \frac{1}{\int dx' \left[\frac{du_0(x')}{dx}\right]^2} \int dx \frac{du_0(x)}{dx} (x-z) S_0(x) \int dx' J(x,x')\, r_0(x').
\end{aligned} \tag{S14}
$$

For Equation (5), we had

$$
\begin{aligned}
\frac{dS_1(t)}{dt}(x-z)S_0(x) &= -\frac{1}{\tau_1}S_0(x) - \frac{1}{\tau_1}S_1(t)(x-z)S_0(x) + \alpha\left[Q_0(x) + Q_1(t)(x-z)Q_0(x)\right] \\
&\qquad \times f_S\left[r_0(x) + \frac{2u_0(x)u_1(t)}{B}\frac{du_0(x)}{dx}\right] \\
&= -\frac{1}{\tau_1}S_0(x) - \frac{1}{\tau_1}S_1(t)(x-z)S_0(x) \\
&\quad + \alpha\left[Q_0(x) + Q_1(t)(x-z)Q_0(x)\right] \\
&\qquad \times \left\{f_S\left[r_0(x)\right] + f_S'\left[r_0(x)\right]\frac{2u_0(x)u_1(t)}{B}\frac{du_0(x)}{dx}\right\} \\
&= \cancel{-\frac{1}{\tau_1}S_0(x) + \alpha Q_0(x)f_S\left[r_0(x)\right]} \\
&\quad - \frac{1}{\tau_1}S_1(t)(x-z)S_0(x) + \alpha Q_1(t)(x-z)Q_0(x)f_S\left[r_0(x)\right] \\
&\qquad + \alpha Q_0(x)f_S'\left[r_0(x)\right]\frac{2u_0(x)u_1(t)}{B}\frac{du_0(x)}{dx} \\
&\quad + \alpha Q_1(t)(x-z)Q_0(x)f_S'\left[r_0(x)\right]\frac{2u_0(x)u_1(t)}{B}\frac{du_0(x)}{dx}.
\end{aligned}
\tag{S15}
$$

By gathering odd terms, we had

$$
\begin{aligned}
\frac{dS_1(t)}{dt}(x-z)S_0(x) &= -\frac{1}{\tau_1}S_1(t)(x-z)S_0(x) + \alpha Q_1(t)(x-z)Q_0(x)f_S\left[r_0(x)\right] \\
&\qquad + \alpha Q_0(x)f_S'\left[r_0(x)\right]\frac{2u_0(x)u_1(t)}{B}\frac{du_0(x)}{dx},
\end{aligned}
\tag{S16}
$$

$$
\begin{aligned}
\frac{dS_1(t)}{dt} &= -\frac{1}{\tau_1}S_1(t) + Q_1(t)\,\alpha\frac{1}{\int dx'\left[(x'-z)S_0(x')\right]^2} \\
&\qquad \times \int dx\,(x-z)S_0(x)(x-z)Q_0(x)f_S\left[r_0(x)\right] \\
&\quad + u_1(t)\frac{2\alpha}{B}\frac{1}{\int dx'\left[(x'-z)S_0(x')\right]^2} \\
&\qquad \times \int dx\,(x-z)S_0(x)Q_0(x)f_S'\left[r_0(x)\right]u_0(x)\frac{du_0(x)}{dx}.
\end{aligned}
\tag{S17}
$$

For Equation (6), we had

$$
\begin{aligned}
\frac{dQ_1(t)}{dt}(x-z)\,Q_0(x) = &-\frac{Q_0(x)+Q_1(t)(x-z)\,Q_0(x)}{\tau_2} \\
&-\alpha\left[Q_0(x)+Q_1(t)(x-z)\,Q_0(x)\right] f_S\left[r(x,t)\right] \\
&+\beta\left[1-Q_0(x)-Q_1(t)(x-z)\,Q_0(x)\right] f_Q\left[I^{\text{tot}}(x,t)\right] \\
= &-\frac{Q_0(x)}{\tau_2}-\alpha Q_0(x)\,f_S\left[r(x,t)\right]+\beta\left[1-Q_0(x)\right] f_Q\left[I^{\text{tot}}(x,t)\right] \\
&-\frac{Q_1(t)}{\tau_2}(x-z)\,Q_0(x)-\alpha Q_1(t)(x-z)\,Q_0(x)\,f_S\left[r(x,t)\right] \\
&-\beta Q_1(t)(x-z)\,Q_0(x)\,f_Q\left[I^{\text{tot}}(x,t)\right] \\
= &-\frac{Q_0(x)}{\tau_2}-\alpha Q_0(x)\,f_S\left[r_0(x)\right]-\alpha u_1(t)\,\frac{2}{B}Q_0(x)\,f_S'\left[r_0(x)\right]u_0(x)\,\frac{du_0(x)}{dx} \\
&+\beta\left[1-Q_0(x)\right] f_Q\left[I_0^{\text{tot}}(x)\right] \\
&+\beta\frac{2}{B}u_1(t)\left[1-Q_0(x)\right] f_Q'\left[I_0^{\text{tot}}(x)\right]\int dx' J\left(x,x'\right)u_0\left(x'\right)\frac{du_0\left(x'\right)}{dx} \\
&-\frac{Q_1(t)}{\tau_2}(x-z)\,Q_0(x)-\alpha Q_1(t)(x-z)\,Q_0(x)\,f_S\left[r_0(x)\right] \\
&-\alpha\frac{2}{B}Q_1(t)\,u_1(t)(x-z)\,Q_0(x)\,f_S'\left[r_0(x)\right]u_0(x)\,\frac{du_0(x)}{dx} \\
&-\beta Q_1(t)(x-z)\,Q_0(x)\,f_Q\left[I_0^{\text{tot}}(x)\right] \\
&-\beta\frac{2}{B}Q_1(t)\,u_1(t)(x-z)\,Q_0(x)\,f_Q'\left[I_0^{\text{tot}}(x)\right] \\
&\times\int dx' J\left(x,x'\right)u_0\left(x'\right)\frac{du_0\left(x'\right)}{dx}. \qquad \text{(S18)}
\end{aligned}
$$

By gathering odd terms, we had

$$\frac{dQ_1(t)}{dt}(x-z)Q_0(x) = -u_1(t)\times\left\{\alpha\frac{2}{B}Q_0(x)f'_S[r_0(x)]u_0(x)\frac{du_0(x)}{dx}\right.$$
$$\left.-\beta\frac{2}{B}[1-Q_0(x)]f'_Q\left[I_0^{\text{tot}}(x)\right]\int dx' J\left(x,x'\right)u_0\left(x'\right)\frac{du_0(x')}{dx}\right\}$$
$$-Q_1(t)\times\left\{\frac{1}{\tau_2}(x-z)Q_0(x)+\alpha(x-z)Q_0(x)f_S[r_0(x)]\right.$$
$$\left.+\beta(x-z)Q_0(x)f_Q\left[I_0^{\text{tot}}(x)\right]\right\}, \tag{S19}$$

$$\frac{dQ_1(t)}{dt} = -u_1(t)\times\frac{1}{\int dx'\left[(x'-z)Q_0(x')\right]^2}\int dx(x-z)Q_0(x)$$
$$\times\left\{\alpha\frac{2}{B}Q_0(x)f'_S[r_0(x)]u_0(x)\frac{du_0(x)}{dx}\right.$$
$$\left.-\beta\frac{2}{B}[1-Q_0(x)]f'_Q\left[I_0^{\text{tot}}(x)\right]\int dx' J\left(x,x'\right)u_0\left(x'\right)\frac{du_0(x')}{dx}\right\}$$
$$-Q_1(t)\times\frac{1}{\int dx'\left[(x'-z)Q_0(x')\right]^2}\int dx(x-z)Q_0(x)$$
$$\times\left\{\frac{1}{\tau_2}(x-z)Q_0(x)+\alpha(x-z)Q_0(x)f_S[r_0(x)]\right.$$
$$\left.+\beta(x-z)Q_0(x)f_Q\left[I_0^{\text{tot}}(x)\right]\right\}. \tag{S20}$$

In summary, we had

$$\frac{d}{dt}\begin{pmatrix} u_1(t) \\ S_1(t) \\ Q_1(t)\end{pmatrix} = \begin{pmatrix} M_{uu} & M_{uS} & 0 \\ M_{Su} & M_{SS} & M_{SQ} \\ M_{Qu} & 0 & M_{QQ}\end{pmatrix}\begin{pmatrix} u_1(t) \\ S_1(t) \\ Q_1(t)\end{pmatrix}, \tag{S21}$$

where

$$M_{uu} = -\frac{1}{\tau_{\rm s}}\Bigg\{1 - \frac{2}{B} \times \frac{1}{\int dx' \left[\frac{du_0(x')}{dx}\right]^2} \times \int dx \frac{du_0\left(x\right)}{dx}\left[1+S_0\left(x\right)\right]\int dx' J\left(x,x'\right)u_0\left(x'\right)\frac{du_0\left(x'\right)}{dx}\Bigg\}, \tag{S22}$$

$$M_{uS} = \frac{1}{\tau_{\rm s}}\frac{1}{\int dx' \left[\frac{du_0(x')}{dx}\right]^2}\int dx \frac{du_0\left(x\right)}{dx}\left(x-z\right)S_0\left(x\right)I_0^{\rm tot}\left(x\right), \tag{S23}$$

$$M_{Su} = \frac{2\alpha}{B}\frac{1}{\int dx' \left[\left(x'-z\right)S_0\left(x'\right)\right]^2}\int dx \left(x-z\right)S_0\left(x\right)Q_0\left(x\right)f_S'\left[r_0\left(x\right)\right]u_0\left(x\right)\frac{du_0\left(x\right)}{dx}, \tag{S24}$$

$$M_{SS} = -\frac{1}{\tau_1}, \tag{S25}$$

$$M_{SQ} = \alpha\frac{1}{\int dx' \left[\left(x'-z\right)S_0\left(x'\right)\right]^2}\int dx \left(x-z\right)S_0\left(x\right)\left(x-z\right)Q_0\left(x\right)f_S\left[r_0\left(x\right)\right], \tag{S26}$$

$$M_{Qu} = -\frac{1}{\int dx' \left[\left(x'-z\right)Q_0\left(x'\right)\right]^2}\int dx \left(x-z\right)Q_0\left(x\right)\Bigg\{\alpha\frac{2}{B}Q_0\left(x\right)f_S'\left[r_0\left(x\right)\right]u_0\left(x\right)\frac{du_0\left(x\right)}{dx} - \beta\frac{2}{B}\left[1-Q_0\left(x\right)\right]f_Q'\left[I_0^{\rm tot}\left(x\right)\right]\int dx' J\left(x,x'\right)u_0\left(x'\right)\frac{du_0\left(x'\right)}{dx}\Bigg\}, \tag{S27}$$

$$M_{QQ} = -\frac{1}{\tau_2} - \frac{1}{\int dx' \left[\left(x'-z\right)Q_0\left(x'\right)\right]^2}\int dx \left(x-z\right)Q_0\left(x\right) \times \Bigg\{\alpha\left(x-z\right)Q_0\left(x\right)f_S\left[r_0\left(x\right)\right] + \beta\left(x-z\right)Q_0\left(x\right)f_Q\left[I_0^{\rm tot}\left(x\right)\right]\Bigg\}. \tag{S28}$$

2 SUPPLEMENTARY FIGURE

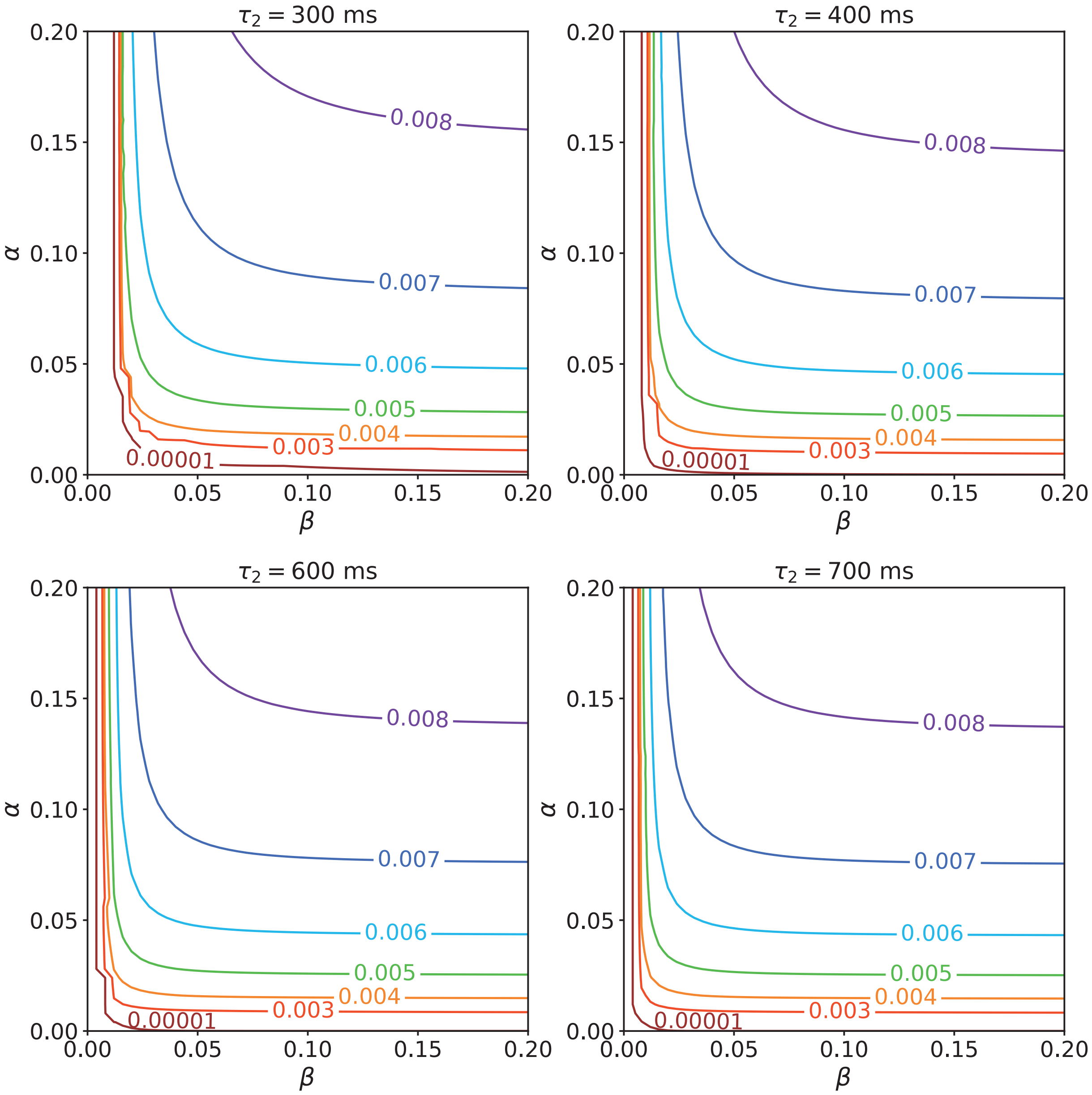

Figure S1. Contour maps of v_{int} when $\tau_2 = 300$ ms, $\tau_2 = 400$ ms, $\tau_2 = 600$ ms, and $\tau_2 = 700$ ms. The intrinsic speeds under these conditions share similar patterns and ranges with those obtained when $\tau_2 = 500$ ms (Figure 6A), indicating the robustness of the intrinsic property of the model to anticipation. The unit of v_{int} is rad/ms. Parameters: $a = 0.5$, $k = 0.5$.
